# NorA, HmpX, and NorB Cooperate to Reduce NO Toxicity during Denitrification and Plant Pathogenesis in *Ralstonia solanacearum*

**DOI:** 10.1128/spectrum.00264-22

**Published:** 2022-04-04

**Authors:** Alicia N. Truchon, Connor G. Hendrich, Adam F. Bigott, Beth L. Dalsing, Caitilyn Allen

**Affiliations:** a Department of Plant Pathology, University of Wisconsin-Madison, Madison, Wisconsin, USA; b Microbiology Doctoral Training Program, University of Wisconsin-Madison, Madison, Wisconsin, USA; Pennsylvania State University

**Keywords:** denitrifying respiration, iron metabolism, nitrosative stress, oxidative stress, plant defenses, plant pathogen

## Abstract

Ralstonia solanacearum, which causes bacterial wilt disease of many crops, requires denitrifying respiration to survive in its plant host. In the hypoxic environment of plant xylem vessels, this pathogen confronts toxic oxidative radicals like nitric oxide (NO), which is generated by both bacterial denitrification and host defenses. R. solanacearum has multiple distinct mechanisms that could mitigate this stress, including putative NO-binding protein (NorA), nitric oxide reductase (NorB), and flavohaemoglobin (HmpX). During denitrification and tomato pathogenesis and in response to exogenous NO, R. solanacearum upregulated *norA*, *norB*, and *hmpX*. Single mutants lacking *ΔnorB*, *ΔnorA*, or *ΔhmpX* increased expression of many iron and sulfur metabolism genes, suggesting that the loss of even one NO detoxification system demands metabolic compensation. Single mutants suffered only moderate fitness reductions in host plants, possibly because they upregulated their remaining protective genes. However, *ΔnorA/norB*, *ΔnorB/hmpX*, and *ΔnorA/hmpX* double mutants grew poorly in denitrifying culture and *in planta*. It is likely that the loss of *norA*, *norB*, and *hmpX* is lethal, since the methods used to construct the double mutants could not generate a triple mutant. Functional aconitase activity assays showed that NorA, HmpX, and especially NorB are important for maintaining iron-sulfur cluster proteins. Additionally, plant defense genes were upregulated in tomatoes infected with the NO-overproducing *ΔnorB* mutant, suggesting that bacterial detoxification of NO reduces the ability of the plant host to perceive the presence of the pathogen. Thus, R. solanacearum’s three NO detoxification systems each contribute to and are collectively essential for overcoming metabolic nitrosative stress during denitrification, for virulence and growth in the tomato, and for evading host plant defenses.

**IMPORTANCE** The soilborne plant pathogen Ralstonia solanacearum (*Rs*) causes bacterial wilt, a serious and widespread threat to global food security. *Rs* is metabolically adapted to low-oxygen conditions, using denitrifying respiration to survive in the host and cause disease. However, bacterial denitrification and host defenses generate nitric oxide (NO), which is toxic and also alters signaling pathways in both the pathogen and its plant hosts. *Rs* mitigates NO with a trio of mechanistically distinct proteins: NO-reductase (NorB), predicted iron-binding (NorA), and oxidoreductase (HmpX). This redundancy, together with analysis of mutants and *in-planta* dual transcriptomes, indicates that maintaining low NO levels is integral to *Rs* fitness in tomatoes (because NO damages iron-cluster proteins) and to evading host recognition (because bacterially produced NO can trigger plant defenses).

## INTRODUCTION

Ralstonia solanacearum (*Rs*), a soil-dwelling plant pathogen, causes bacterial wilt disease in a wide range of economically important plants, including tomatoes. Bacterial wilt is a serious socioeconomic problem in tropical regions, especially in developing countries where crop loss can be devastating for subsistence farmers (1). To date, there is no effective control strategy to combat bacterial wilt (2). *Rs* draws on its broad repertoire of metabolic capabilities to survive in soil and water, invade plant roots, and colonize and obstruct its host’s water-transporting xylem vessels (3). The pathogen’s metabolism adapts rapidly as it transitions among diverse microniches in surface water, in soil, and inside hosts (4–6). Plant xylem vessels, the primary in-host habitat of *Rs*, contain little oxygen but have substantial levels of nitrate (NO_3_), around 30 mM (7).

Bacteria have several ways to make ATP under low-oxygen conditions. These include fermentation and respiration using alternate terminal electron acceptors (TEAs), such as sulfur, iron, and nitrogen (8). Nitrate respiration and denitrification require a series of membrane-bound and periplasmic enzymes which reduce NO_3_^-^ stepwise to dinitrogen gas (N_2_) (9). Denitrifying respiration allows organisms to produce energy from NO_3_^-^ in hypoxic environments such as soil, marine sediments, landfills, wastewater treatment plants, bioreactors, and the inside of eukaryotic hosts (9–16).

**FIG 1:**
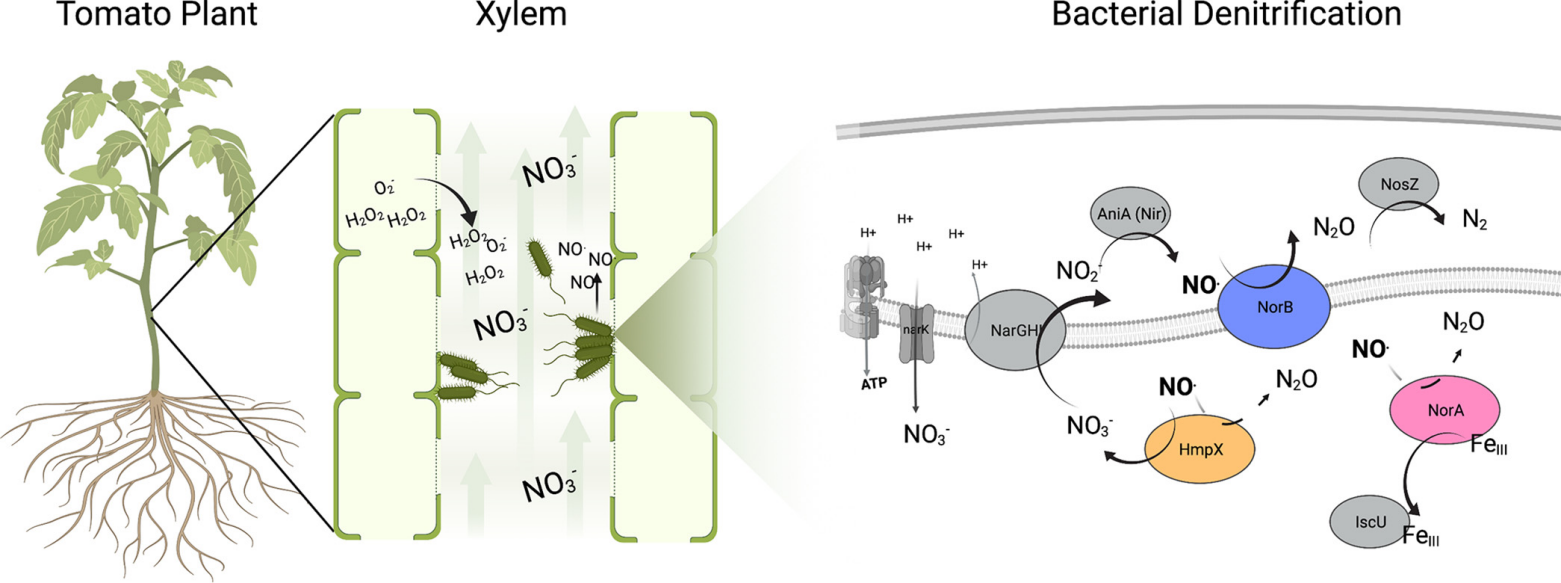
Ralstonia solanacearum strain GMI1000 denitrifies in tomato xylem, generating energy and NO. Tomato plant xylem contains ∼30 mM NO_3_^-^. *Rs* uses denitrifying respiration to reduce NO_3_^-^ and generate ATP. Denitrification reduces NO_3_^-^ to N_2_ gas via the enzymes NarGHI, AniA, NorB, and NosZ. NO_3_^-^ reduction generates toxic NO that must be oxidized, reduced, or sequestered to prevent cellular damage. In response to bacterial infection, tomato plants also produce oxidative compounds, including H_2_O_2_, and O_2_^-^. R. solanacearum NorA, NorB, and HmpX proteins interact with NO by either reducing NO to N_2_O, oxidizing NO to NO_3_^-^, or binding NO to protect iron centers which are vulnerable to damage from NO. Figure created in part using BioRender.

Nitrate metabolism is broadly conserved across plant pathogenic *Ralstonia* spp. (17–19). *Rs* strain GMI1000 has a complete pathway for denitrifying respiration, wherein the nitrate reductase NarG reduces NO_3_^-^ to NO_2_^-^ (nitrite), the nitrite reductase AniA reduces NO_2_^-^ to NO (nitric oxide), the nitric oxide reductase NorB converts NO to N_2_O (nitrous oxide), and finally the nitrous oxide reductase NosZ converts N_2_O to N_2_ (Fig. 1). When *Rs* invades tomato stems, xylem oxygen levels decline even further and the pathogen’s denitrification genes are substantially upregulated (7, 20). We previously established that *Rs* uses NO_3_^-^ and its reduction products as TEAs to generate proton motive force, which drives ATP synthesis (7). Possibly as a result, denitrification contributes quantitatively to *Rs* growth *in planta* and bacterial wilt virulence (7, 20).

However, denitrifying respiration comes at a cost. The pathway generates two highly reactive nitrogen species (RNS): NO_2_^-^ and NO (9, 16). NO is especially toxic because it binds to the ferrous iron centers of important iron-sulfur cluster (Fe-S) proteins like aconitase, destroying enzymatic activity (21, 22). NO is a source of both nitrosative stress (i.e., the covalent addition of NO to an atom) and reactive oxygen species (ROS), because NO interacts with itself or other oxygen or nitrogen species to form even more damaging potent oxidant species like peroxynitrite (23). These secondary NO products damage crucial cellular components, including metalloproteins, lipids, and nucleic acids (23, 24). We will collectively refer to these effects as nitrosative stress.

In addition to the NO generated by prokaryotic respiration, microbes encounter NO produced by their eukaryotic hosts (25, 26). NO can act as a diffusible signal that does not require a carrier, and it is a major plant signaling molecule which rapidly regulates plant defense functions, including cell death (27, 28). Many hosts also produce NO and H_2_O_2_ to directly kill pathogens (29–32). In response to this oxidative attack, animal and plant pathogens, including *Erwinia* spp., *Pseudomonas* spp., Staphylococcus aureus, and Neisseria gonorroheae, use denitrification pathway enzymes like NO_2_^-^ reductase (NIR) and NO reductase (NOR); not only to produce energy, but also to reduce the toxic load of RNS, sometimes by decoupling them from the electron transport chain (9, 13). Microbes have evolved additional specialized mechanisms to mitigate RNS stress (7, 9, 30, 31, 33–36). The flavohemoglobin Hmp is an oxidoreductase that uses a globin-like NO-binding domain, NAD, and FAD to catalyze the conversion of NO and O_2_ to NO_3_^-^ when oxygen is available, or to reduce NO to N_2_O in the absence of O_2_ (29, 31, 37, 38). Homologs of Hmp are present across the bacterial domain (29, 30, 39). A second protective mechanism involves NO-binding di-iron proteins. Some di-iron proteins have a putative repair of iron centers (RIC) function which is thought to use the conserved hemerythrin-like domain to directly bind NO and/or interact with damaged iron clusters to mitigate cellular damage caused by NO; the exact mechanism of this remains uncertain (40–43).

The *Rs* GMI1000 genome has genes encoding a putative di-iron NO-binding protein, NorA; a NO reductase, NorB; and an oxidoreductase, HmpX. When *Rs* grows in tomato xylem, *norA*, *norB*, and *hmpX* are upregulated 75-, 51-, and 43-fold, respectively, relative to levels during *Rs* growth in rich medium (20). These genes were all highly expressed and moreover, were among the most differentially expressed genes *in planta*, where *Rs* cells experience an oxidative environment (20, 32, 44). This upregulation implied that during plant pathogenesis, *Rs* depends on the products of *norA*, *norB*, and *hmpX* to mitigate the nitrosative stress produced by its own denitrifying respiration and by the plant host. This functional redundancy suggested that detoxifying NO is critically important for R. solanacearum. We tested this hypothesis using a panel of single and double mutants lacking *norA*, *hmpX*, and *norB* combined with transcriptomic, biochemical, and plant assays.

## RESULTS

### *norA*, *norB*, and *hmpX* are upregulated in denitrifying cultures and by exogenous NO.

The proteins encoded by *norA*, *norB*, and *hmpX* in *Rs* strain GMI1000 are conserved across diverse bacteria, including environmental isolates and plant and animal pathogens (Table S1A in the supplemental material). Furthermore, all three were encoded in the genomes of the several hundred sequenced strains in the R. solanacearum species complex. Previous functional analyses have demonstrated that *Rs norB* encodes a NO reductase and *hmpX* encodes an oxidoreductase (7). We identified locus Rsp 0958 as *norA* because its product resembles known proteins with hemerythrin-like domains that bind NO to reduce cellular nitrosative stress (40). It is most similar to NorA from C. necator (73% amino acid [AA] identity), and to YtfE from Salmonella enterica and DnrN from N. gonorrhoeae (∼50% AA identity). All three of these proteins have been implicated in nitrosative stress mitigation (Table S1A) (40, 45, 46). *Rs* NorA, NorB, and HmpX each contain the highly conserved heme- or globin metal cofactor-binding domains necessary to reduce NO toxicity (Fig. S1 in the supplemental material). These genomic analyses suggested that *Rs* NorA, NorB, and HmpX might all contribute to mitigating NO damage.

**FIG 2:**
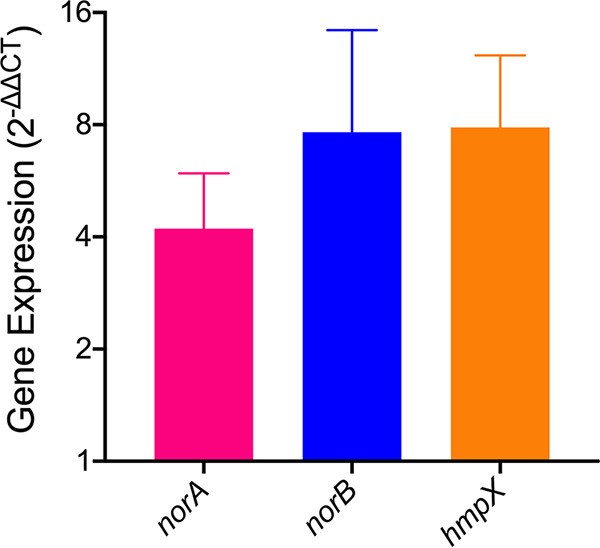
Exogenous NO induces expression of *norA*, *norB*, and *hmpX*. Relative gene expression of wild-type R. solanacearum GMI1000 as determined by qRT-PCR. RNA was extracted from R. solanacearum cells cultured for 16 h under denitrifying conditions (VDM + 30 mM NO_3_^-^ with 0.1% O_2_), then treated with 1 mM NO donor sodium nitroprusside for 3 h in denitrifying conditions. Gene expression is shown relative to untreated R. solanacearum cells. Wild-type gene expression for *norA*, *norB*, and *hmpX* was normalized to *rplM.* Data are mean +/– standard error of the mean (SEM) (*norA*, *P* = 0.0211; *norB*, *P* = 0.0552; *hmpX*, *P* = 0.0255, one-sample *t* test). Data are the means of 4 biological experiments, each containing 3 technical replicates. Fold change was calculated using the 2^-ΔΔ^*^CT^* (cycle threshold) method.

A previous transcriptomic analysis found that when *Rs* grows in the stressful plant host environment, it upregulates *norA*, *norB*, and *hmpX* by 75-, 51-, and 43-fold, respectively, relative to their expression in rich medium (20, 32, 44). Indeed, these were among the genes most differentially expressed *in planta* (Table S1B). *norA*, *norB*, and *hmpX* were also highly expressed in denitrifying *Rs* cells cultured at 0.1% O_2_, a condition that produces an oxidative environment. Treating denitrifying cultures with exogenous NO further increased expression of *norA* (5-fold, *P* = 0.0211, one sample *t* test), *norB* (9-fold, *P* = 0.0552), and *hmpX* (8-fold, *P* = 0.0255) (Fig. 2). We used sodium nitroprusside (SNP) as the NO donor for these experiments because NO release from SNP occurs over a longer period of time and is neither temperature- nor pH-dependent (47). However, like all NO donors, SNP has limitations; in this case, the potential for light-triggered release of hydrogen cyanide. Given that endogenous and exogenous NO have similar effects, it is more likely that the upregulation of *norA*, *norB*, and *hmpX* genes is associated with the NO-binding functions of NorA, NorB, and HmpX than with cyanide toxicity. The significant upregulation of *norA*, *norB*, and *hmpX* in the oxidative plant environment, and in response to this exogenous NO donor, is consistent with the hypothesis that these genes are important for NO metabolism.

To explore whether these three genes were all under the control of the same NO-responsive regulator, we used RegPrecise to find predicted binding sites (48). Binding sites for the NO-responsive Rrf2 family regulator NsrR were present at the 5′-ends of *norB* and *hmpX*, but not at that of *norA*. Upstream of *norA*, we found a binding site for NorR, the predicted NO-inducible, sigma-54 dependent Fnr family regulator. This suggested that these genes are under different regulons. In *Rs*, NsrR is predicted to have nine genes in its regulon, but NorR is predicted to regulate only the *norAR* operon (48, 49). However, NorR regulates both *norA* and *norB* in other bacteria, such as the closely related C. necator (49–52). To confirm the bioinformatic prediction that *Rs* NorR exclusively regulates *norA*, we measured the expression of *norA*, *norB*, and *hmpX* in a *ΔnorR* deletion mutant. Indeed, when *Rs ΔnorR* grew under denitrifying conditions, *norA* expression was reduced 15-fold relative to that of the wild-type parent strain, while expression of *norB* and *hmpX* did not change (Fig. S2A). This indicates that *norB* and *hmpX* are not regulated by NorR, and that the *Rs* response to NO is complex and involves at least one additional regulatory mechanism (Fig. S2B). This finding prompted us to investigate the functional interplay of NorA, NorB, and HmpX.

### Δ*norA*, Δ*norB*, and Δ*hmpX* mutants upregulate genes for iron and sulfur metabolism in denitrifying conditions.

**FIG 3:**
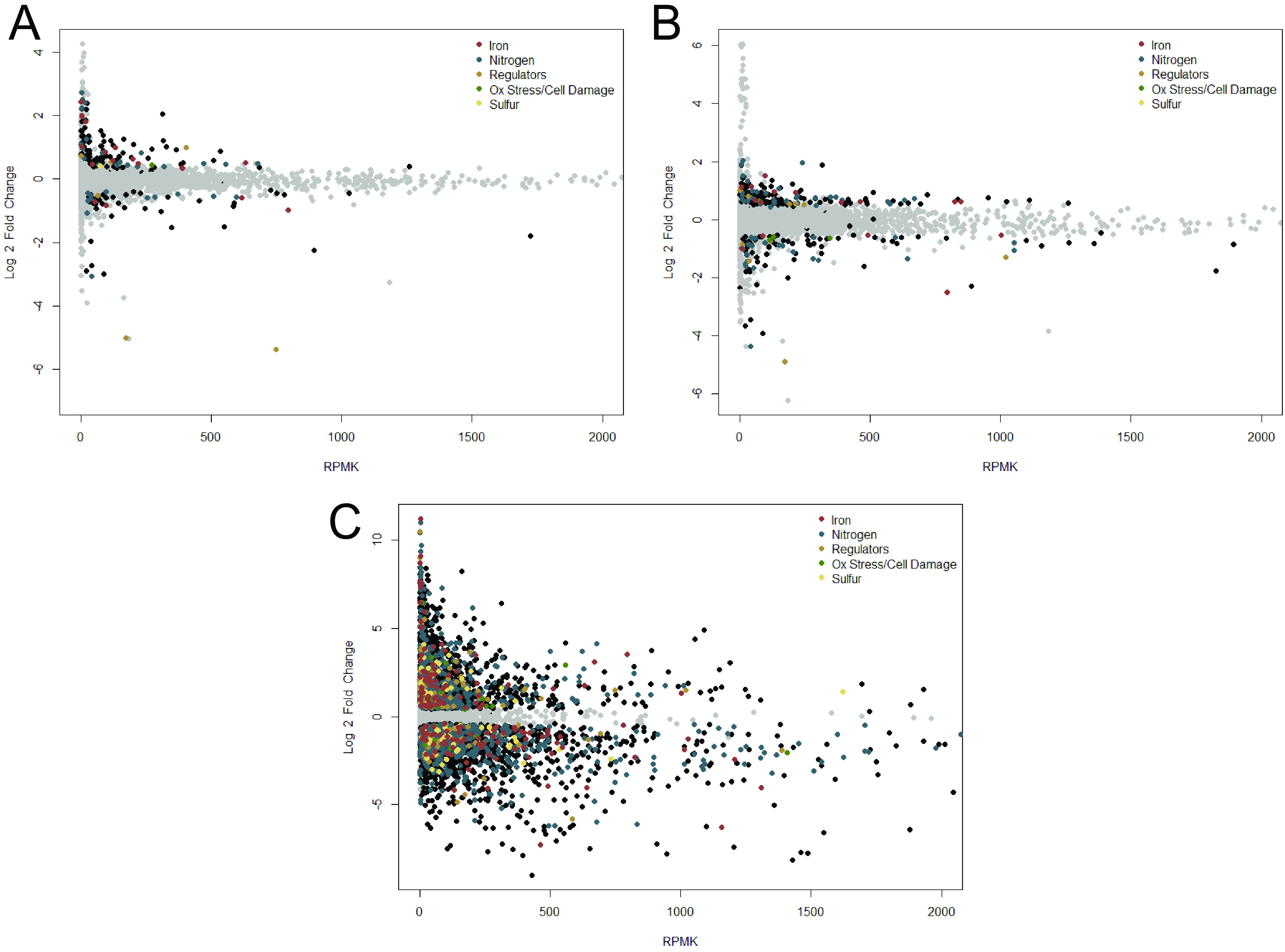
Transcriptomic profiles of R. solanacearum Δ*norA*, Δ*norB*, and Δ*hmpX* mutants relative to wild type. Plots showing gene expression in Δ*norA* (A), Δ*norB* (B), and Δ*hmpX* (C). RNA was extracted and sequenced from bacteria after 16 h growth in denitrifying conditions (VDM + 30 mM NO_3_^-^ with 0.1% O_2_). Log2-fold change in expression is plotted against reads per million per kilobase (RPMK) to show change in regulation versus transcript abundance, relative to gene expression in wild-type R. solanacearum strain GMI1000. Differentially expressed genes (defined as *P* < 0.05,) are shown in black, except for genes with GO terms related to iron metabolism (red), nitrogen metabolism (blue), regulators (brown), oxidative stress and cellular damage (green), sulfur metabolism (yellow). Genes not differentially expressed are shown in gray. Of the 6,108 open reading frames in the R. solanacearum genome, Δ*norA* had 187 DEGs, Δ*hmpX* had 281 DEGs, and Δ*norB* had 4,112 DEGs compared to wild-type cells.

Oxidative molecules like NO cause nitrosative stress that damages cellular components, including Fe-S proteins, lipids, and DNA, leading to the general bacterial stress (SOS) response (39, 53, 54). We hypothesized that cells lacking the putative stress mitigation genes *norA*, *norB*, or *hmpX* would suffer nitrosative damage. Such damage could be reflected in altered expression of genes encoding iron, sulfur, and repair pathways. We tested this hypothesis by profiling the transcriptomes of wild-type, Δ*norA*, Δ*norB*, and Δ*hmpX* strains after 16 h growth in denitrifying conditions, a time point at which NO_3_^-^ respiration generates NO and nitrosative stress. All three mutations substantially affected the *Rs* transcriptional profile. Relative to wild-type *Rs*, the Δ*norA* and Δ*hmpX* mutants had 187 and 281 differentially expressed genes (DEGs), respectively. A surprising 2/3 of the genome, or 4,105 of 6,200 open reading frames (ORFs), were differentially expressed in the Δ*norB* mutant (Fig. 3).

Many of the 187 DEGs in the Δ*norA* mutant were upregulated and predicted to be involved in stress tolerance, iron acquisition, and inorganic nitrogen metabolism (Fig. 3A). Among the most upregulated DEGs were the iron homeostasis regulator *fur2*; Rsp0415, encoding the putative iron-stress response sigma-factor RpoE; and Rsp0421, putatively encoding RhbC, a component of siderophore synthesis. Among the most abundantly expressed DEGs were *narG* and *narH*, encoding subunits of a nitrate reductase; and Rsc0754, encoding putative peroxidase AhpC. In *ΔnorA*, *hmpX* was slightly downregulated 1.96-fold (*P* = 3.21E–5) and *norB* expression was not significantly different from that in the wild type (WT), although it was already in the wild-type strain’s top 10 most abundantly expressed genes (Table S2). Overall, this transcriptomic profile suggests that loss of the predicted NO-binding protein NorA causes increased nitrosative stress which affects iron metabolism, but the *ΔnorA* mutant mitigates this by upregulating genes for a wide range of protective mechanisms.

In the Δ*hmpX* mutant, about half of the 281 DEGs were upregulated and were related to inorganic nitrogen or sulfur metabolism (Fig. 3B). Among the most highly upregulated genes were *nsrR*, encoding a nitrate sensitive repressor; *hsdM* (Rsc3396) and *hsdR* (Rsc3384), encoding a putative type I restriction modification system; and *sbp*, encoding a sulfate binding protein involved in cysteine synthesis. Although *norB* was slightly downregulated in *ΔhmpX* (1.61-fold, *P* = 1.29E–5) and *norA* expression was not significantly different from that of the wild type, both genes remained in the top 20 most abundantly expressed genes, and *norB* was the single most abundant gene transcript expressed by Δ*hmpX* in denitrifying conditions (Table S1B). This profile suggests that Δ*hmpX* is still metabolizing NO and may pivot its metabolic strategies to acquire more sulfur to address damage to iron, sulfur, or Fe-S cluster proteins.

Loss of the NO reductase NorB had the most dramatic transcriptional effect. Genes involved in iron metabolism, sulfur metabolism, or cellular repair were most highly upregulated (Fig. 3C). The top 3 most upregulated genes, all encoding iron acquisition proteins, were upregulated over 1,000-fold (*P* < 3.34E–67). Even the regulator *fur2* was upregulated 854-fold (*P* = 6.26E–89). The *ΔnorA* and *ΔhmpX* transcriptomes showed similar trends, but with a smaller magnitude than in *ΔnorB* (Fig. 3C). In addition, *ΔnorB* significantly upregulated *norA* and *hmpX* by 2.43-fold (*P* = 1.38E–9) and 11.67-fold (*P* = 1.9E–58), respectively (Table S1B).

The global upregulation of iron homeostasis regulators like *fur2* in the Δ*norA* and Δ*norB* mutants indicated damage to Fe-S cluster proteins, but Δ*hmpX* and Δ*norB* also upregulated error-prone DNA polymerase *dnaE2* 1.62-fold (*P* = 0.029) and 118.05-fold (*P* = 2.18E–32), respectively, suggesting that cells lacking *hmpX* or *norB* also experience oxidative damage to DNA.

**FIG 4:**
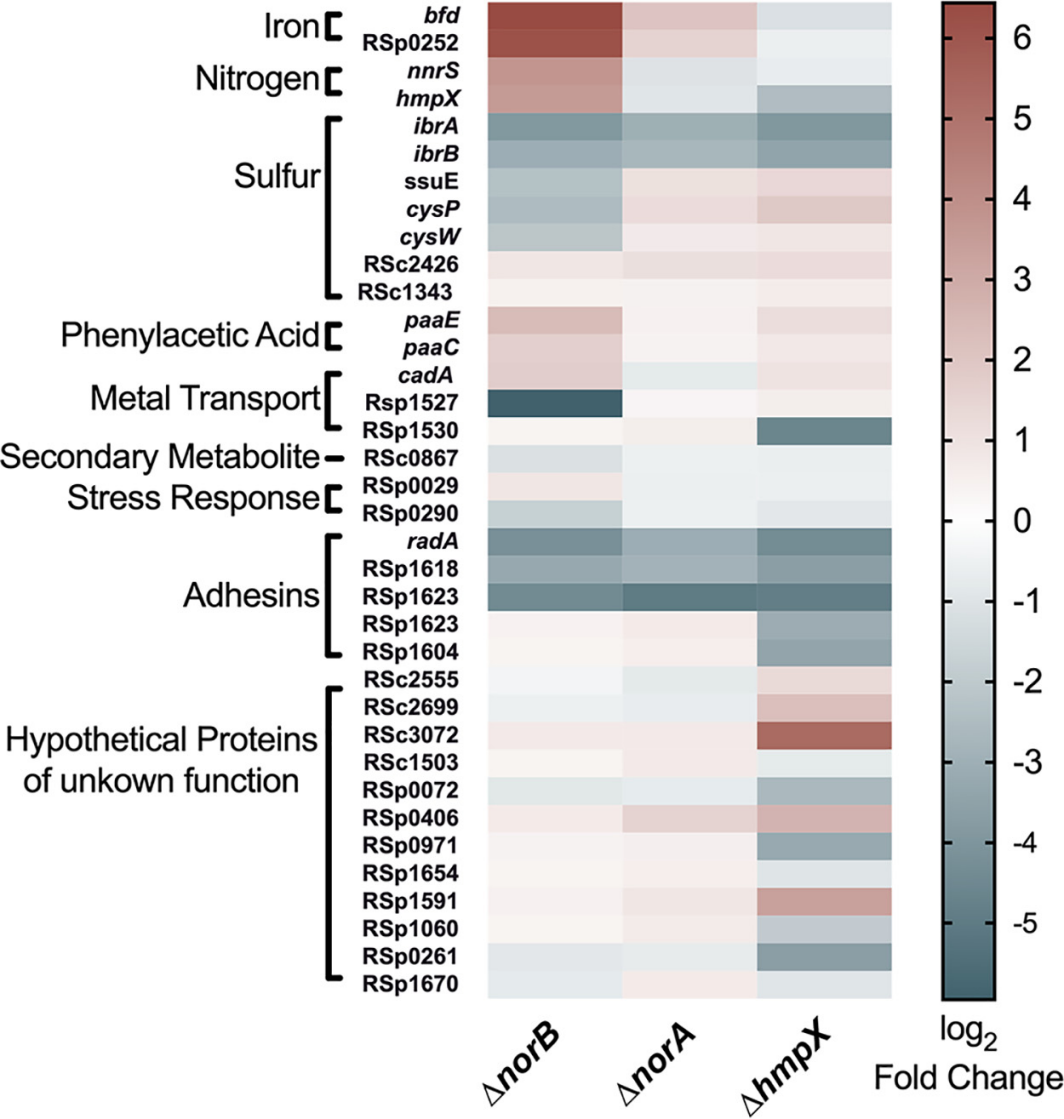
R. solanacearum mutants lacking NorA, NorB, and HmpX differentially expressed some of the same genes. Heat map showing up- or downregulation (relative to wild-type strain GMI1000) of selected differentially expressed genes (DEGs) from all three mutants. RNA was extracted and sequenced from bacteria after 16 h growth in denitrifying conditions (VDM + 30 mM NO_3_^-^ with 0.1% O_2_). For all three mutants, genes differentially expressed relative to wild-type were compared, and shared DEGs were joined with SQL. Known or putative function was used to sort genes into categories indicated on the left. Log2-fold change is represented as a heat map showing expression of selected shared DEGs relative to expression levels to wild-type cells. Red indicates upregulated genes, white indicates genes not significantly different from wild-type, and blue indicates downregulated genes, as shown in the scale bar at right.

More broadly, mutants lacking either *norA*, *norB*, or *hmpX* shared 43 common DEGs, 21 of which have known homologs or domains with predicted function (Fig. 4). All three mutants differentially expressed bacterioferritin-encoding *bfd* and seven genes related to sulfur metabolism. Further, all three mutants upregulated *paaE*, which is predicted to encode degradation of phenylacetic acid (PAA) or a plant auxin growth hormone, which could interact with plant hosts. Interestingly, the most-downregulated genes for all three mutants were in the Rsp1617-1623 operon (about 10- to 30-fold, *P* < 0.021949), which is predicted to be involved in cell attachment. Together, these shared DEGs suggest that all three mutants suffered enough RNS to cause detectable cellular damage.

### A mutant lacking *norB* accumulates NO in culture and has severely reduced virulence *in planta*.

**FIG 5:**
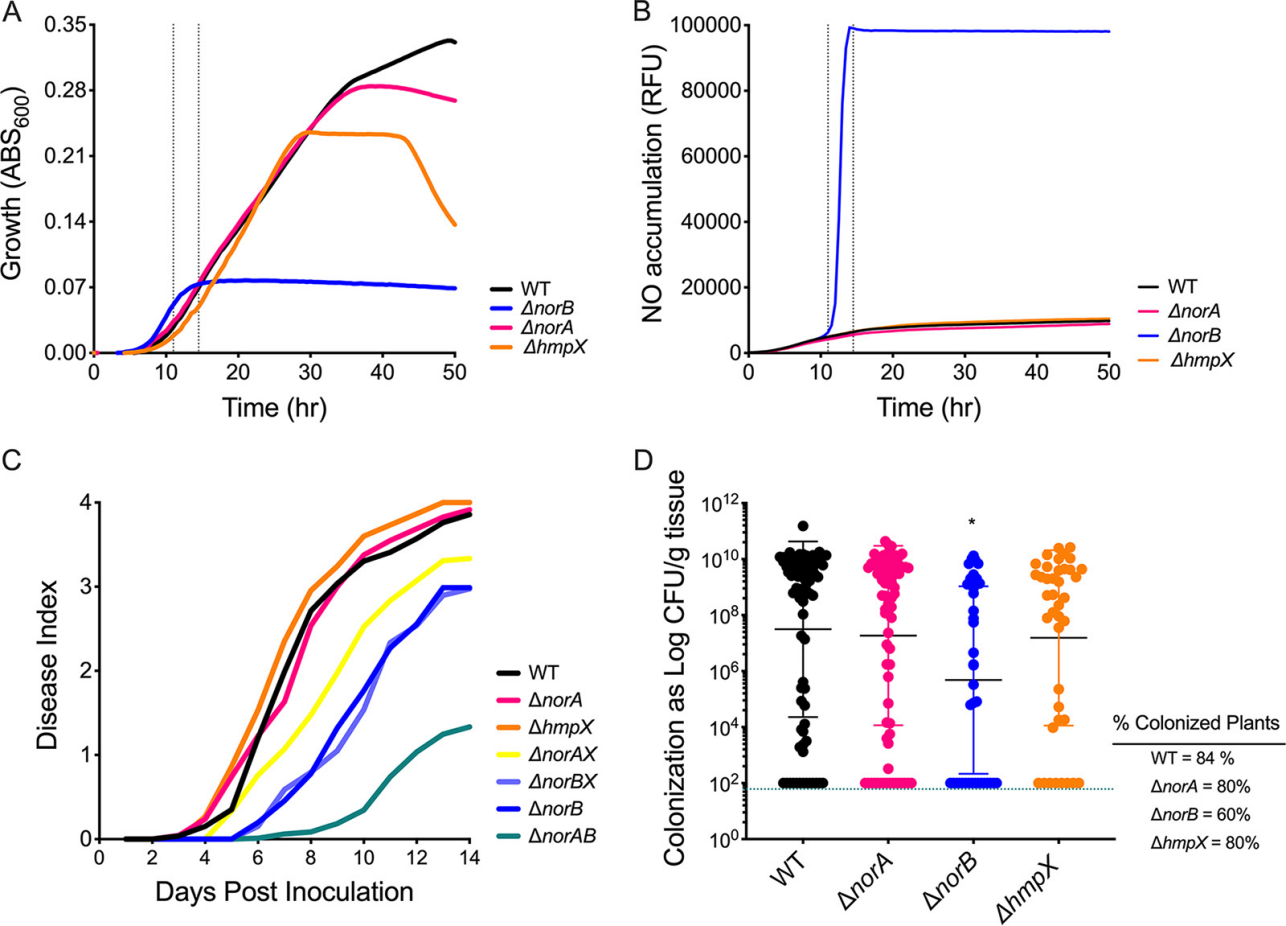
Behavior of R. solanacearum Δ*norA*, Δ*norB*, and Δ*hmpX* mutants in denitrifying culture conditions and *in planta*. (A) Growth of wild type and mutant R. solanacearum cells in denitrifying conditions (VDM + 30 mM NO_3_^-^ with 0.1% O_2_) in shaking 96-well plates, shown as Abs_600_. Data are mean +/– SEM data and are mean of 4 biological experiments, each with 3 technical replicates. Gray bars represent time of toxic NO accumulation ∼12 h. (B) Accumulation of nitric oxide (NO) over time in the cultures in panel A, measured as relative fluorescence units using the NO-specific fluorescent indicator DAF-FM-DA. Excitation and emission measured at 495/515 nm. Data are the means of 4 biological experiments, each with 3 technical replicates. Gray bars represent time of toxic NO accumulation, ∼12 h. (C) Bacterial wilt disease progress of 16-day-old, wilt-susceptible ‘Bonny Best’ tomato plants following naturalistic soil-soak inoculation with 1 × 10^8^ CFU wild-type or mutant *Rs* cells. Plants were assessed for wilt symptoms on a scale of 0 to 4 over 14 days, with 4 indicating completely wilted plants. Data shown represent the mean disease index of 40 to 93 plants per treatment in 3 to 6 biological replicates. Virulence of the *ΔnorB*, *ΔnorAB*, *ΔnorAX*, and *ΔnorBX* mutants was lower than that of wild-type strain GMI1000, while *ΔhmpX* was slightly more virulent than wild type (*P* = 0.0017, 0.0016, 0.0037, 0.0020, and 0.0009, respectively, repeated measures analysis of variance [ANOVA]). (D) R. solanacearum population sizes in tomato midstems 4 days after 2 × 10^6^ CFU of R. solanacearum were applied to the cut petiole of the first true leaf. Bacterial populations were quantified by grinding and serial-dilution plating stem cross-sections. Each dot shows the *Rs* population from one plant, with 40 to 80 plants per treatment across 3 to 4 biological replicates. Horizontal bars indicate geometric means of population sizes; values for stem samples below the detection limit (100 CFU/g) were entered as 100; asterisks (*) indicate that mean population size is different from that in the wild type (*P* = 0.0049, Kruskal-Wallis test). For each strain, the percentage of samples containing detectable *Rs* cells is indicated under “% Colonized Plants” on the right.

Transcriptomic analysis suggested that *norA*, *norB*, and *hmpX* are important for mitigating the RNS stress that *Rs* experiences during denitrifying respiration in culture and in the low-oxygen plant host xylem (7, 20). We directly tested this hypothesis by assessing in-culture and *in planta* behaviors of *Rs* deletion mutants lacking *norA*, *norB*, or *hmpX* (Fig. 5).

In aerobic culture, where *Rs* does not denitrify, the Δ*norA*, Δ*norB*, and Δ*hmpX* strains grew as well as parent strain GMI1000 (Fig. S3). None of the three mutants grew as well as the wild type in hypoxic denitrifying culture, although their growth was affected to differing degrees (Fig. 5A). For the first 24 h, Δ*norA* and Δ*hmpX* grew like wild type, but their growth plateaued at ∼36 and ∼28 h, respectively, while wild type did not enter stationary-phase until ∼48 h. Growth of the Δ*norB* mutant under denitrifying conditions plateaued much earlier, at ∼12 h, while wild type was still in early log-phase growth. The limited growth of the *ΔnorB* mutant was consistent with the development of toxic conditions that interfered with bacterial growth.

To directly test whether these three mutants accumulate NO, we used the NO-specific fluorescent probe DAF-FM-DA to measure NO accumulation over time in denitrifying cultures (Fig. 5B). The ∼12 h growth plateau of the Δ*norB* mutant correlated exactly with a rapid accumulation of NO in the culture, which contained at least 10 times more NO than the wild-type cultures. Wild-type, Δ*norA*, and Δ*hmpX* cells did not accumulate detectable amounts of NO, likely because NorB reduces NO almost as fast as it is produced in all three strains.

We previously determined that Δ*norB* has a virulence defect and that neither *ΔnorB* nor Δ*hmpX* colonize tomato plants as well as the wild-type strain following a naturalistic soil-soak inoculation (7). To see if loss of *norA* also affected these behaviors, we inoculated tomato plants with either Δ*norA*, Δ*norB*, or Δ*hmpX*. The *ΔnorB* mutant caused significantly reduced bacterial wilt symptoms in the soil-soak assay (Fig. 5C). By 72 h after tomato stems were directly inoculated through a cut leaf petiole, the *ΔnorB* population in tomato midstems was around 2 orders of magnitude smaller in size than that of the wild type (Fig. 5D). In contrast, neither the Δ*norA* nor the *ΔhmpX* mutants differed significantly from the wild type with respect to bacterial wilt virulence or stem colonization after petiole inoculation. Results of these *in planta* experiments are consistent with the finding that the Δ*norB* mutant accumulates toxic levels of NO which severely impair its growth in denitrifying culture. In contrast, the Δ*norA* and Δ*hmpX* mutants functioned much like the wild type in both conditions. The *in planta* defects of *ΔnorB* are likely explained by the mutant’s inability to detoxify the NO generated by denitrifying respiration during plant pathogenesis. These defects further suggest that without either NorA or HmpX, *Rs* can overcome the nitrosative stress produced by bacterial denitrification and the plant host, likely by changing the transcription of iron and sulfur metabolism genes. However, despite massive transcriptomic changes, *Rs* cannot compensate for loss of the NorB nitric oxide reductase, as evidenced by the mutant’s loss of virulence, plant colonization defects, and reduced fitness in culture.

### NorA, NorB, and HmpX function together in denitrifying culture.

Detoxification of reactive radical species like NO is critically important for the fitness of denitrifying bacteria (55–57). Although Δ*norA* and Δ*hmpX* single mutants had wild-type virulence and were only modestly reduced in late-stage denitrifying growth compared to wild-type *Rs*, their transcriptional signatures indicated that they did suffer RNS stress early in denitrifying cultures. Additionally, during denitrification, the Δ*norB* mutant strongly upregulated expression of *norA* and *hmpX*. We wondered how *Rs* would behave in the absence of two or more components of its RNS mitigation system.

**FIG 6:**
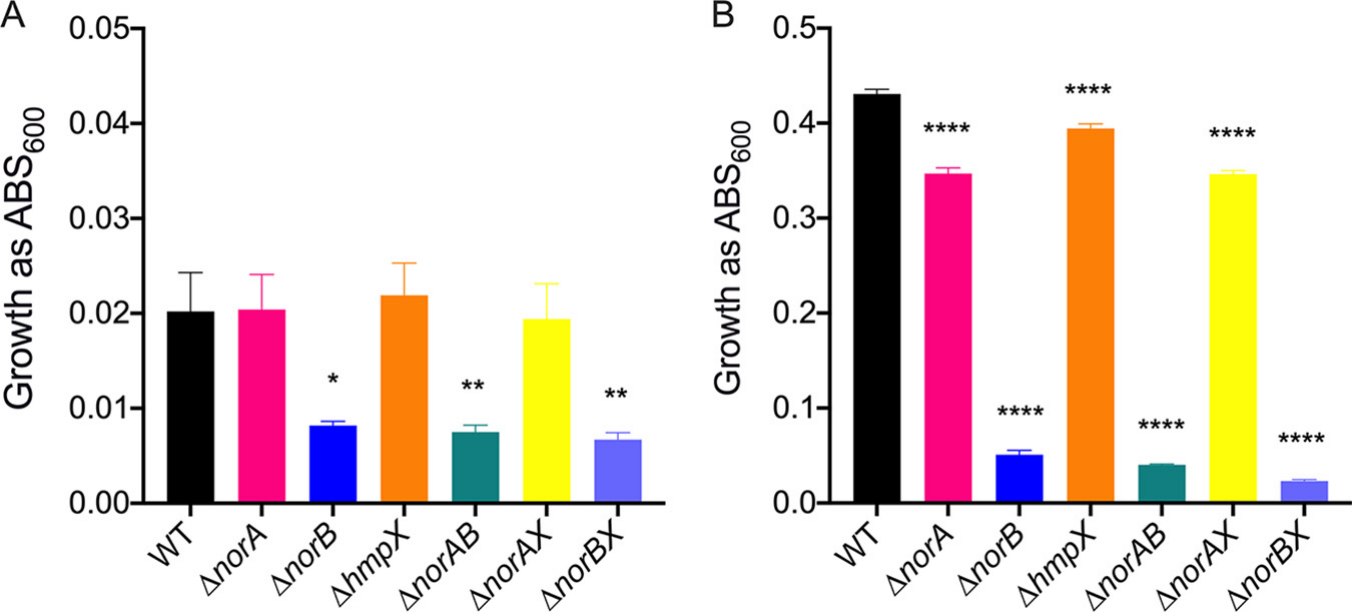
The NorB nitric oxide reductase is important for R. solanacearum growth in denitrifying conditions. Growth of wild type, Δ*norA*, Δ*norB*, Δ*hmpX*, and double mutants in VDM + 30 mM NO_3_- with 0.1% O_2_ shaking in 96-well plates, measured spectrophotometrically as absorbance at 600 nm, after (A) 16 h culture or (B) 36 h culture. Data shown reflect the mean +/– SEM of 4 biological replicate experiments, each containing 3 technical replicates. For both 16 h and 36 h, asterisks indicate difference in growth from that of the wild-type strain: *, *P* < 0.05; **, *P* < 0.008; ****, *P* < 0.001 (one-way ANOVA).

We therefore created double-deletion mutants lacking multiple genes; *norA* and *norB* (*ΔnorAB)*; *norA* and *hmpX* (*ΔnorAX*); *hmpX* and *norB* (*ΔnorBX*). Persistent efforts to use the same methods to create a *ΔnorA/norB/hmpX* triple mutant were unsuccessful, suggesting that the loss of all three proteins is lethal to *Rs*. After 16 h of growth under denitrifying conditions (corresponding to the time RNA was harvested for transcriptional analysis), the Δ*norAX* double mutant grew as well as the wild type. However, both double mutants lacking *norB* grew to lower endpoints (yield) than WT, Δ*norA*, or Δ*hmpX* (*P* < 0.0078, analysis of variance [ANOVA]), although the growth of Δ*norAB* and Δ*norBX* was not significantly different from that of the Δ*norB* single mutant (Fig. 6A). After 36 h under denitrifying conditions, all single and double mutants had significantly lower absorbance at 600 nm (Abs_600_) than the wild type. Further, single and double mutants lacking NorB were dramatically reduced in growth at 36 h (Fig. 6B). Cells of the Δ*norAB* and Δ*norBX* double mutants grew only around 10% as much as the wild-type, Δ*norA*, or Δ*hmpX* cells (*P* < 0.001, ANOVA). Additionally, these double mutants also reached a 35% lower Abs_600_ reading than the Δ*norB* single mutant (*P* < 0.001, ANOVA). These cumulative growth differences show that the nitric oxide reductase NorB plays an irreplaceable role in mitigating NO stress both early and late in denitrifying growth in culture. However, the putative NO-binding protein NorA and the oxidoreductase HmpX also protect *Rs* when NO accumulates, especially during later stages of denitrifying metabolism. To see if the Δ*norAB*, Δ*norAX*, or Δ*norBX* double mutants were also reduced in plant virulence, we inoculated tomato plants with these three double mutants. All three caused significantly less disease than the wild-type strain (Fig. 5C). While the *ΔnorAX* mutant did worse than either the Δ*norA* or Δ*hmpX* single mutants (*P* = 0.0006 and 0.0008, respectively, repeated measures ANOVA), the Δ*norBX* mutant performed similarly to the Δ*norB* mutant, and the Δ*norAB* double mutant caused significantly less disease than the Δ*norB* mutant (*P* = 0.0108, repeated measures ANOVA). Additionally, the Δ*norAB* double mutant colonized plants worse than the wild type but did not significantly differ from the Δ*norB* single mutant (Fig. S5).

### *norA*, *norB*, and *hmpX* contribute to cellular protection from nitrosative stress.

Denitrifying metabolism damages iron-sulfur (Fe-S) cluster proteins, such as the TCA cycle enzymes fumarase and aconitase, by binding to iron and changing the oxidative state of the bound catalytic center (58). *Rs* mutants which lacked *norA*, *norB*, or *hmpX* altered the expression of many genes involved in iron and sulfur metabolism, which suggested that these mutants experienced damage to Fe-S proteins and would be more susceptible to nitrosative stress.

To test the hypothesis that *Rs* mutants lacking NorA, NorB, or HmpX are more susceptible to nitrosative stress, we treated denitrifying cultures with exogenous NO or H_2_O_2_ at 16 and 36 h, then measured their growth recovery (Fig. S4). At 16 h, all tested strains recovered similarly from exposure to the NO donor spermine-NONOate (Fig. S4A). At 36 h, the Δ*norB* mutant actually recovered from NO treatment better than all other strains (*P* < 0.0001, ANOVA) (Fig. S4B). Similarly, the Δ*norAB*, *ΔnorAX*, and *ΔnorBX* double mutants were more tolerant of H_2_O_2_ than the wild type at 16 h (Fig. S4C), although their recoveries did not differ at 36 h (Fig. S4D). We concluded that single or double mutants lacking *norA*, *norB*, or *hmpX* were not more susceptible to the levels of exogenous oxidative stress tested under these conditions.

**FIG 7:**
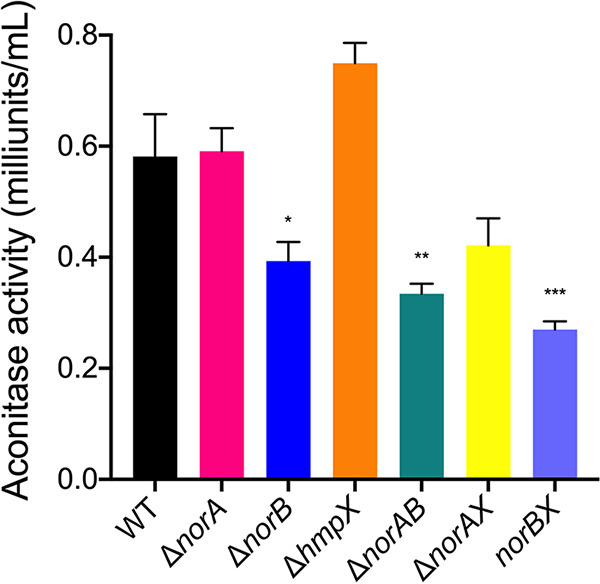
R. solanacearum needs NorB to prevent damage to the iron-sulfur protein aconitase. R. solanacearum wild type, Δ*norA*, Δ*norB*, Δ*hmpX*, and double mutants were cultured for 36 h in VDM + 30 mM NO_3_- with 0.1% O_2_. For each strain, activity of the abundant iron-sulfur protein aconitase was measured using an Aconitase Activity assay kit (Sigma-Aldrich) according to the manufacturer’s instructions. Data shown reflect the mean +/– SEM of 3 biological replicates. Asterisks indicate difference from growth of the wild-type strain: *, *P* = 0.0360; **, *P* = 0.0057; ***, *P* = 0.0008 (ANOVA).

As a measure of Fe-S cluster damage, we quantified aconitase activity in various *Rs* strains growing in denitrifying conditions, normalizing enzyme activity to cell density to account for differences in growth between strains. After 16 h of culture, wild-type and all mutant cells contained similar aconitase levels (data not shown). However, by 36 h, all strains lacking *norB* had reduced aconitase activity compared to that of wild-type cells (Fig. 7). While the wild-type strain contained an average of 0.58 milliunits/mL, Δ*norB*, Δ*norAB*, Δ*norBX* produced 0.39, 0.33, and 0.27 milliunits/mL of active aconitase, respectively (*P* = 0.0360, 0.0057, and 0.008, respectively, ANOVA). Aconitase activity in Δ*norAB* and Δ*norBX* double mutants trended lower than that in the *ΔnorB* single mutant, although there were no significant differences. At 0.42 milliunits/mL, aconitase activity in the Δ*norAX* mutant similarly trended down but was not significantly different from that of the wild type. Together with the transcriptional profiles suggesting that *ΔnorA* and *ΔhmpX* experience iron and sulfur stress, these trends indicate that NorA and HmpX help to protect Fe-S proteins, including aconitase. However, NorB is the major source of *Rs* cellular protection in denitrifying conditions, as evidenced by both transcriptional and direct enzyme analyses.

### Bacterially produced NO affects plant host transcriptional responses.

**FIG 8:**
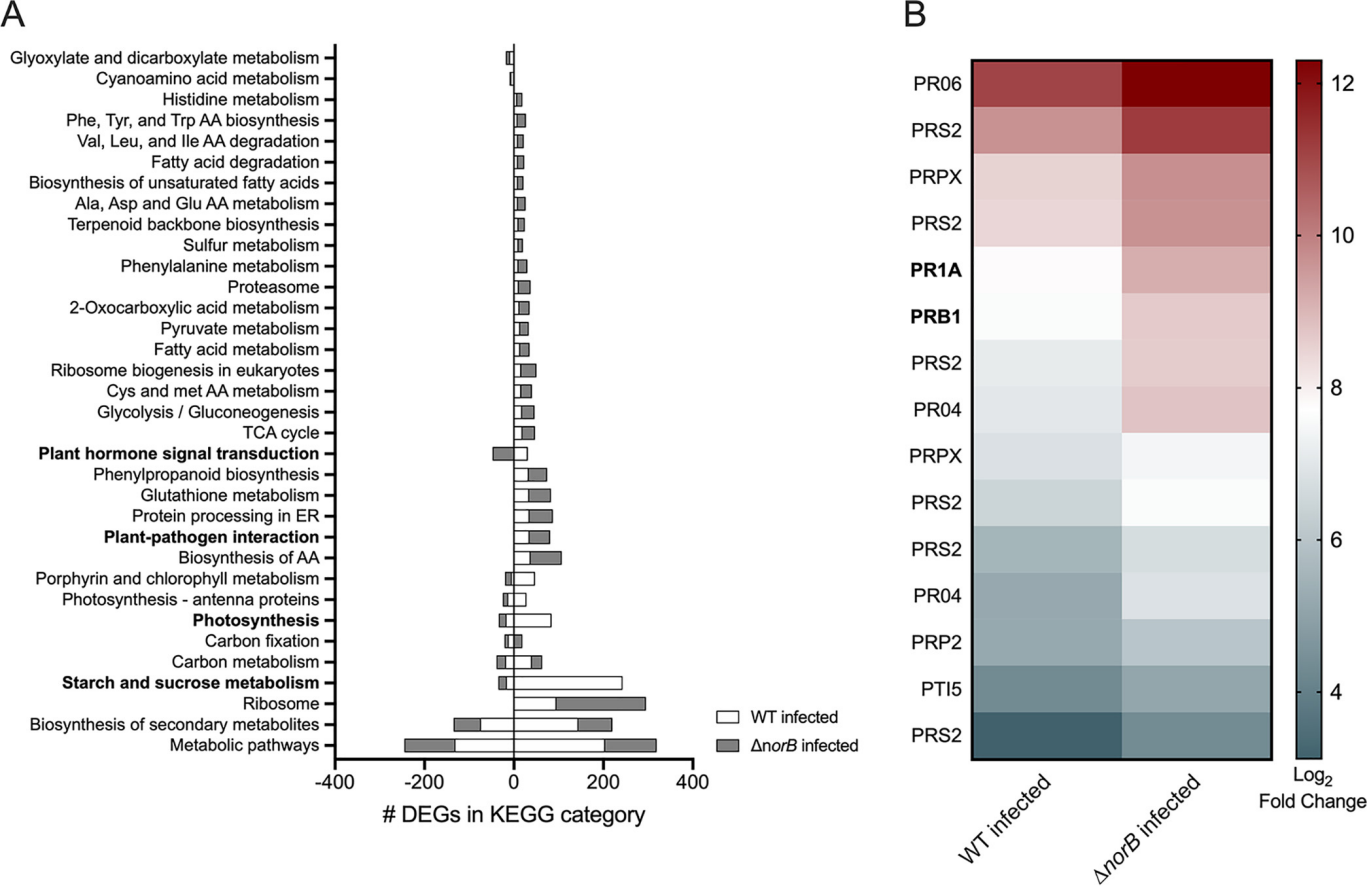
Transcriptomic response of tomato plants infected with either R. solanacearum wild-type strain GMI1000 or the *ΔnorB* mutant. RNA was harvested and sequenced from stems of ‘Bonny Best’ tomato plants 72 h after they were petiole-inoculated with 2 × 10^6^ CFU of either wild-type or Δ*norB* R. solanacearum, or with water. RNA from water-treated (healthy) plants served as the control to determine differential gene expression. (A) Differentially expressed tomato genes (DEGs) were sorted into 34 KEGG categories shared by both wild-type- and Δ*norB*-infected plants. Numbers of DEGs in each KEGG category from wild-type- or Δ*norB*-infected plants are graphed in stacked columns for comparison. The *x* axis shows the number of DEGs assorted into the indicated KEGG pathways, as determined using KOBAS software by NovoGene. For details on identification of DEGs, see the Methods section. (B) Expression levels of 15 pathogenesis-related genes in plants infected with either wild-type or Δ*norB* R. solanacearum cells. Pathogenesis-related DEGs were identified by searching the Uniprot annotations (https://www.uniprot.org/blast/) of all DEGs for wild-type- and Δ*norB*-infected plants for any genes annotated with the terms “pathogen,” “pathogenesis,” or “biotic interaction.”

Having shown that nitrosative stress is toxic to *Rs* cells both *in planta* and in culture, we investigated ways by which ROS could affect bacterial-plant interactions. NO is a free radical signaling molecule that affects every stage of the plant life cycle (28, 59, 60). In particular, NO interacts with plant hormones to change signaling pathways during plant growth and biotic interactions (28, 59, 61). We hypothesized that increasing the amount of NO produced by the pathogen would alter plant perception of *Rs* during infection. We tested this by comparing the transcriptomes of tomato plants infected with either wild-type *Rs* or the NO-accumulating Δ*norB* mutant to the transcriptomes of healthy plants. As expected, in response to infection by either wild-type or *ΔnorB Rs*, tomato plants significantly changed gene expression patterns, including pathways in the KEGG and Gene Ontology (GO) categories of general cellular metabolism and processes involved in plant-pathogen interactions. (Fig. 8, Fig. S6, Table S4). Differentially expressed genes fell into 39 KEGG categories in plants infected with wild type *Rs* and 42 categories in Δ*norB*-infected plants, with 34 KEGG categories shared by plants infected with either strain. Overall, Δ*norB* induced about twice as many DEGs in tomatoes as wild type *Rs* (Fig. 8A). Most DEGs in plants infected with either wild-type or Δ*norB* mutant cells changed expression of basic metabolic pathways, biosynthesis of secondary metabolites, plant-pathogen response, and plant hormone signal transduction. Wild-type *Rs* induced more DEGs involved in tomato starch and sucrose metabolism and photosynthesis. While wild-type *Rs* induced plant hormone signal transduction, Δ*norB* mutant cells suppressed plant hormone signal transduction. Wild-type and Δ*norB* mutant uniquely expressed plant DEGs in 5 and 8 KEGG categories, respectively (Fig. S6A). Specifically, wild-type cells upregulated host plant nitrogen metabolism and carotenoid biosynthesis, while Δ*norB* cells induced biosynthesis of arginine and alkaloids.

Most strikingly, plants infected with *ΔnorB* differentially upregulated all the pathogen response (PR) genes annotated with the KEGG terms pathogen, biotic, and defense, including the salicylic acid and ethylene pathway defense signaling genes, PR1a and PR1b, which were previously validated as contributing to tomato resistance to bacterial wilt (Fig. 8B). Together, the KEGG and GO-term analyses of tomato DEGs showed that plants had different transcriptional responses to infection by wild-type and Δ*norB Rs*. In particular, the tomato host mounted stronger defenses against the NO-overproducing Δ*norB* mutant, possibly because the higher NO levels activated plant defense signaling pathways.

## DISCUSSION

Few bacteria can compete in the low-nutrient, low-oxygen niche of plant xylem vessels, but R. solanacearum (*Rs*) thrives in xylem, partly because it can respire on nitrate. The disadvantage of this metabolic strategy is that it generates potentially toxic levels of highly reactive NO as a byproduct. In addition, *Rs* cells in xylem confront ROS and RNS released by plant defenses (32, 62). Our goal was to determine how this pathogen protects itself from the resulting NO toxicity and nitrosative stress (7, 20). These mechanisms have been well studied in human pathogens, but little is known about how plant pathogenic bacteria mitigate the damaging effects of oxidative conditions they encounter in their hosts (32, 44).

Many bacteria accomplish this task with nitric oxide reductases (NORs) like NorB, flavorubredoxin oxido-reductases like HmpX, and di-iron proteins like NorA (63). *Rs* homologs of all three of these proteins were well conserved at the amino acid level, notably at residues which bind cofactors.

The NorA hemerythrin-like domain includes the histidine residues needed to bind the iron cofactor, which are likely responsible for its NO-binding activity (58). We found that *norA*, but not *norB* or *hmpX*, is regulated by the NO-inducible transcriptional regulator NorR. Transcriptomic analysis of a *ΔnorR* mutant indicated that *norA* is the only protein-encoding gene in the NorR regulon; this is noteworthy, because NorR typically also regulates *norB* and/or *hmpX* (49, 51). The roles and regulation of NorA homologs have been studied in some human pathogens but have not been considered in a plant pathogen (9, 29, 40).

NorB contains a large, well-conserved heme-oxidase domain responsible for NO reductase activity; this domain had homology to many other NOR proteins (13). Single-subunit membrane-bound NORs like NorB are typically tied to the electron transport chain and generate ATP (13). However, rapid accumulation of NO in the Δ*norB* mutant made it impossible to distinguish the phenotypic effects of energy loss from those of NO toxicity, or to experimentally determine whether NorB contributes to ATP generation in *Rs*.

HmpX, which requires O_2_ for its NO oxidase activity, can also reduce NO in anoxic conditions. The fact that HmpX contains highly conserved residues in both the globin-like NO-binding domain and the FAD/NAD-binding domains needed for full oxidoreductase activity suggests that *Rs* denitrifies or encounters RNS stress in both microaerobic and anoxic conditions (38). Both conditions occur in the xylem vessels of *Rs*-infected plants (7). In addition to encountering low oxygen in plant hosts, *Rs* likely experiences low-oxygen denitrifying conditions in soil during its saprophytic life between plant hosts. Many soil-dwelling microbes depend on nitrate respiration and denitrification to thrive in highly variable soil microenvironments (64).

Taken together, the high conservation of these three protective proteins, not only in *Rs*, but also in other pathogens that do not contain the full denitrification pathway, such as the enteric pathogens E. coli and S. enterica, suggests they are important for pathogen-host interactions, possibly to mitigate oxidative host defenses (65–67).

Transcriptomic analysis of *Rs* during denitrification revealed that NO damage globally changes the bacterium’s gene expression. NorB and HmpX were recently shown to help *Rs* colonize tomato plants, but it was unclear whether they contribute to *in planta* fitness because they mitigate nitrosative stress. Wild-type cells treated with NO strongly upregulated *norA*, *norB*, and *hmpX*, suggesting an important role in nitrosative stress response. Furthermore, *Rs* mutants lacking these three genes had transcriptional signatures consistent with nitrosative stress. In denitrifying conditions, all three mutants upregulated iron and sulfur metabolism to varying degrees. However, the Δ*norA*, Δ*norB*, and Δ*hmpX* mutants also had distinct transcriptional profiles and differentially expressed some shared DEGs at different magnitudes, suggesting redundant functionality by distinct mechanisms and a hierarchical importance where NorB > HmpX > NorA.

The most differentially expressed and most abundant gene transcripts in the *norA*, *norB*, *and hmpX* mutants were associated with iron and sulfur metabolism, consistent with the damage to Fe-S proteins caused by accumulated nitrosative stress (39, 68). Because NO is both highly reactive and diffusible, it harms many cellular components and can also interact with S-nitrosylated proteins to change transcription in both the bacterium and the plant (69). The catalytic centers of iron and sulfur proteins are especially susceptible to oxidative damage (70). Common bacterial responses to nitrosative stress and Fe-S damage include upregulation of the iron sulfur cluster biogenesis genes *isc*/*nif*, siderophore biosynthesis and secretion, and general bacterial stress response (SOS) systems (70–72). The transcriptomes of denitrifying *Rs* strains were consistent with this pattern. The Δ*norA* mutant upregulated the key iron sulfur biogenesis operon, including *iscS*/*R* (Rsc1018-1026), and many iron acquisition genes, including the major ferric uptake regulator FUR2 and putative siderophore biosynthesis and receptor proteins, Rsp0419 and Rsp0416. This suggests NorA normally mitigates nitrosative stress by preventing NO damage to iron centers, so in its absence *Rs* cells must acquire more iron to generate new, undamaged Fe-S clusters. The Δ*hmpX* mutant upregulated sulfur metabolism, including *ssuB/E* and *sbp* genes, as well as the error prone DNA polymerase *dnaE2*. Upregulation of sulfur and damage response proteins is consistent with upregulation of sulfur metabolism to regenerate or repair damaged bio-available sulfur in Fe-S centers (73, 74). Alternatively, *ΔhmpX* may acquire more sulfur to repair cysteine, which is commonly destroyed by nitrosative stress (75). Over 2/3 of the *Rs* GMI1000 genome was differentially expressed in the Δ*norB* mutant, which suffered intense nitrosative stress. As observed for *ΔnorA* and Δ*hmpX*, many of this mutant’s most upregulated and most abundantly expressed genes were involved in iron and sulfur metabolism, but *ΔnorB* also upregulated additional damage response pathways. The Δ*norB* mutant transcriptome carries the signatures of substantial NO damage and a nitrosative stress response, consistent with its growth defects in denitrifying culture and *in planta*.

Intriguingly, all three single mutants downregulated a cluster of genes encoding putative collagen-like binding adhesins. These are likely involved in cell-to-cell or cell-to-host attachment. Suppression of adhesion-related proteins suggests the hypothesis that *Rs* cells respond to nitrosative stress by detaching from fellow bacteria or xylem vessel surfaces. Stress-induced detachment could help *Rs* cells escape from dense biofilms where toxic levels of NO accumulate, or from host cells releasing oxidative bursts. All three single mutants also upregulated degradation of the auxin phenylacetic acid, a plant growth hormone; auxins help shape tomato defenses against *Rs* (76, 77). By reducing levels of a plant hormone, *Rs* could change plant signaling, and reduce the oxidative defense response. It would be interesting to determine if a *Δpaa* deletion mutant of *Rs* is less successful in plant hosts.

We previously determined that NorB acts in denitrifying conditions such as those found in xylem, but a mutant lacking this enzyme was as virulent as wild type when it was introduced directly into tomato xylem through a cut leaf petiole (7). However, deleting *norB* did significantly lower *Rs* virulence in a more holistic soil soak inoculation assay that forces the pathogen to find, enter, and colonize unwounded plants through the roots. Reduced Δ*norB* mutant virulence following this naturalistic inoculation method suggests that *Rs* depends on NorB during the plant invasion process. At this point *Rs* cells may be more susceptible to nitrosative stress produced by other *Rs* cells, competing microbes, or by the plant host. Although the Δ*norA* and Δ*hmpX* mutants had wild-type virulence and plant colonization, our *in vitro* experiments confirmed that NorA, NorB, and HmpX are all required for normal growth under denitrifying conditions. Although Δ*norA* and *ΔhmpX* strains suffered only mild growth defects in denitrifying culture, these two proteins may be important for NO detoxification in the microaerobic soil environments where *Rs* survives between plant hosts. It would be interesting to see if the Δ*norA*, Δ*norB*, and Δ*hmpX* mutants survive as well as wild-type *Rs* in low-oxygen soil microcosms.

Growth of single Δ*norA* and Δ*hmpX* mutants in denitrifying culture plateaued earlier than that of wild type and furthermore, these mutants had significant growth defects at 36 h but not 16 h, suggesting these proteins contribute to *Rs* fitness when nitrosative stress accumulated. Under these conditions the Δ*norB* mutant quickly accumulated large amounts of NO, and its growth arrest coincided exactly with spiking NO levels in the culture. The toxic effects of NO likely drove the global gene expression changes observed in the Δ*norB* mutant, which was sampled for transcriptomic analysis after 16 h of culture. These data suggest that at this point Δ*norB* cells were so damaged they were simultaneously trying to repair proteins and synthesize them *de novo*. In an apparent attempt to compensate, the Δ*norB* mutant also upregulated expression of *hmpX* and *norA*, as well as genes for many Fe-S enzymes, including aconitase. Enzyme activity assays confirmed that *Rs* strains lacking *norB* had reduced aconitase activity, a direct indicator of global cellular damage. In contrast, aconitase activity was not significantly lower than wild type in *norA* or *hmpX* single or double mutants. This suggests that cells depend on NorA and HmpX when NorB can no longer reduce the cellular pool of NO. Measuring growth of Δ*norA* and Δ*hmpX* mutants on older plants that have more developed immune systems, larger xylem vessels, and larger populations of denitrifying bacteria where the pathogen experiences more nitrosative stress per cell could reveal if NorA and HmpX make quantitative fitness contributions in late stage disease.

We hypothesized that loss of RNS mitigating proteins would make *Rs* more susceptible to oxidative stress, but on the contrary, all three double mutants trended toward increased ability to recover from treatment with H_2_O_2_. We speculate that because of their defects, these strains were already experiencing enough stress that they were primed to mitigate the inhibitory effects of H_2_O_2_ more effectively than wild-type (78). This is consistent with our previous observation that *Rs* cells isolated directly from the oxidative plant environment have higher tolerance of oxidative and cold stress than *Rs* cells grown *in vitro* (62). Analyzing the transcriptomes of double mutants could reveal if their unexpectedly high stress tolerance is explained by upregulation of genes involved in iron and sulfur metabolism, the SOS response, and other stress repair mechanisms.

Tomato plants responded differently at the transcriptional level to infection with NO-accumulating Δ*norB* mutant than to infection with wild-type *Rs.* Relative to healthy control plants, *ΔnorB* induced more tomato DEGs than the wild type *Rs*. However, plants infected with wild-type *Rs* expressed more starch and sucrose metabolism genes and more genes involved in photosynthesis. This could indicate that during successful infection, *Rs* cells manipulate their plant host to increase the available nutrients. It is theorized that *Rs* forces plants to load sugar into xylem sap, but the mechanism for this is still unknown (79). Alternatively, increased defenses triggered by the *ΔnorB* mutant may reduce photosynthesis as part of the well-established growth versus defense trade-off. It was also interesting that only wild-type *Rs* differentially induced genes in the KEGG category of “nitrogen metabolism.” However, arginine biosynthesis was upregulated exclusively in Δ*norB*-infected plants. Arginine is thought to be involved in plant nitric oxide synthase (NOS) activity, which oxidizes l-arginine to NO and l-citrulline (80). Increased arginine expression by Δ*norB*-infected plants suggests that either the NO accumulated by this mutant is sufficient to change plant signaling and induce NOS, or that the accumulated NO causes a damage response.

Plants can recognize damage-associated molecular patterns (DAMPs, such as cell wall fragments and extracellular non-self DNA) and pathogen-associated molecular patterns (PAMPs, like flagellar proteins and peptidoglycan) (81–83). In response to DAMPs and PAMPs, both plants and animals produce a defensive burst of ROS and RNS such as H_2_O_2_ and NO (84). As discussed above, its strong oxidative properties make NO a potent antimicrobial compound. However, NO is also a key actor in plant defense signaling pathways. Notably, all tomato genes annotated with the terms pathogen, biotic, and defense were expressed at higher levels in plants infected with *ΔnorB*. This heightened defense suggests that bacterially produced NO made the *Rs* cells more visible to plants and could be one reason why the Δ*norB* mutant suffers reduced virulence. We speculate that in addition to protecting itself from oxidative damage, *Rs* may also reduce NO levels in order to hide from its plant hosts. It would be interesting to measure defense responses and bacterial wilt disease susceptibility in plants pretreated with exogenous NO. If high NO levels can alter plant signal transduction, NO-treated plants would have broadly enhanced disease resistance.

## MATERIALS AND METHODS

### Bacterial growth conditions.

The R. solanacearum and Escherichia coli strains used are listed in Table S5. E. coli strains were grown in LB broth and R. solanacearum strains were grown on rich casamino acid-peptone-glucose (CPG) medium at 28°C, shaking at 225 rpm unless otherwise noted. As appropriate, antibiotics were used at the following concentrations: 25 μg/mL kanamycin and 10 μg/mL tetracycline. We grew bacteria under the previously determined denitrifying conditions: in VandenMooter denitrifying medium (VDM) modified with 30 mM NO_3_ in low oxygen (either 0 or 0.1% O_2_), with shaking at 225 rpm, or on medium speed in a microplate reader (BioTek, Winooski, VT, USA) (7). For hypoxic assays (0.1% O_2_), bacteria were grown in an anaerobic chamber (InvivO2, Baker Ruskinn, Sanford, ME, USA) set to 0.1% O_2_. For anoxic assays (0% O_2_), cells were grown in GasPak pouches (Becton Dickinson, Franklin Lakes, NJ, USA). After pouches were opened to add the reagents, a fresh anaerobic sachet was added to immediately restore anaerobic conditions.

### Mutant construction.

All *Rs* mutants were constructed in phylotype I sequevar 18 strain GMI1000. Unmarked Δ*norA* and Δ*norR* mutants lacking the complete *norA* or *norR* ORF were generated using Gibson assembly and *sacB* positive selection vector pUFR80 as described (79, 85). Briefly, PCR with KapaHifi DNA-polymerase was used to amplify up- and downstream regions of Rsp0958 (*norA*) or Rsp0959 (*norR*); PCR fragments were annealed with pUFR80 to form either pUFR80-*norA* or pUFR80-*norR*, which were then transformed into GMI1000; kanamycin and sucrose selection were used to generate clean in-frame deletion mutants. Double mutants were made by transforming previously constructed plasmids into the Δ*norA* or the previously constructed Δ*norB* and Δ*hmpX* mutant backgrounds (7). All mutants were confirmed with sequencing. All primers and mutant strains are listed in Table S5.

### Plant experiments.

Disease assays were conducted as previously described (86). Briefly, wilt-susceptible cv. ‘Bonny Best’ tomato plants were grown at 28°C with a 12 h day-night light cycle and watered daily with 0.5× strength Hoagland’s solution (87). Two-week-old seedlings were transplanted into 4-inch pots containing ∼80 g potting mix. Two days later, unwounded plants were inoculated by drenching the soil with 50 mL of a 1 × 10^8^ CFU/mL bacterial suspension. Inoculum was determined turbidometrically and confirmed by dilution plating as described (88). Plant wilt symptoms were rated using a 0 to 4 disease index for 14 days (88).

To assess bacterial colonization, 0.100 g stem tissue was ground at 2,200 rpm for 1.5 min, 2 times with a 4 min rest to cool between cycles, in a Powerlyzer (Qiagen, Hilden, Germany). Samples were serial-dilution plated on CPG medium and colonies were counted after 48 h.

### Alignments.

The NCBI BLASTp nonredundant protein sequence database (https://blast.ncbi.nlm.nih.gov) was used to compare percent amino acid identity (% AA ID) and percent query cover (% QC) of R. solanacearum NorA, NorB, and HmpX to C. necator, N. gonorrhoeae, S. aureus, E. coli, S. enterica, and X. fastidiosa. The MUSCLE multiple sequence alignment tool (https://www.ebi.ac.uk/Tools/msa/muscle) was used to align R. solanacearum NorA, NorB, and HmpX amino acid sequences with homologs in C. necator, N. gonorrhoeae, S. aureus, E. coli, S. enterica, and X. fastidiosa.

### RNA extraction and transcriptomic analyses.

**(i) RNA extraction.** RNA was collected from denitrifying R. solanacearum bacterial cultures or from the stem tissues of plants 72 h after petiole-inoculation with *Rs* strain GMI1000, *ΔnorB Rs*, or water.

For transcriptomes of cultured cells, bacteria were grown in triplicate in VDM + 30 mM NO_3_^-^ without shaking for 16 h at 28°C in 0.1% O_2_. Subsamples were dilution-plated to determine CFU/mL, then samples were centrifuged at room temperature for 5 min at 3,000 × *g*, supernatant was removed, and pellets were frozen in liquid nitrogen. Total RNA was extracted using a modified version of the Quick-RNA MiniPrep Kit (Zymo Research, Irvine, CA, USA). Briefly, frozen pellets were resuspended in 400 μL cold Tris-EDTA (TE, pH 8) with 1 mg/mL lysozyme, 0.25 μL Superase inhibitor (Ambion, Austin, TX, USA), and 80 μL of a 10% SDS solution oxidation-fermentation (OF) 10% SDS, vortexed for 10s, then transferred to a new 2-mL tube and shaken at 300 rpm for 2 min. An 800-μL volume of RNA lysis buffer was added, then samples were cleaned according to the kit manufacturer’s instructions. Samples were eluted in 100 μL nuclease-free water, then DNA was removed using the DNA-free DNase kit, according to the manufacturer’s instructions for Rigorous DNase treatment (Invitrogen, Carlsbad, CA, USA). After DNase inactivation, samples were further cleaned by chloroform extraction and precipitated overnight at –20°C with 100 μM sodium acetate (pH 5.5) and 66% ethanol. Samples were checked for concentration on a Nanodrop (Thermo Fisher Scientific, Wilmington, DE, USA), for DNA contamination by PCR using the reverse transcription-quantitative PCR (qRT-PCR) primers serC_F/R, and for RNA integrity (RIN) using an Agilent Bioanalyzer 21000 (Agilent, Santa Clara, CA, USA). All sequenced samples had RIN values above 7.3 (89).

For dual plant-pathogen transcriptomes *in planta*, samples were harvested 21 days after susceptible cultivar ‘Bonny Best’ tomatoes were inoculated with ∼2,000 CFU of each bacterial strain through the cut petiole. At 72 h after inoculation, approximately 0.1 g stem tissue was collected from the site of inoculation, immediately frozen in liquid nitrogen, and stored at –80°C. Another 0.1 g of tissue was collected from directly below the inoculation site and ground in bead beater tubes using a PowerLyzer (Qiagen, Hilden, Germany) for two cycles of 2,200 rpm for 90 s, with a 4 min rest between cycles. This material was then dilution-plated to measure bacterial colonization. Total RNA was then extracted from stem samples colonized with between 10^8^ and 10^9^ CFU/g of tissue, using a hot-phenol chloroform method (20). Between 4 and 5 individual plants were pooled per biological replicate. Nucleic acid sample quality was checked using a Nanodrop, Agilent Bioanalyzer, and qRT-PCR primers actin_F/R (89). All samples had RIN values above 7.2.

All RNA samples were sent to Novogene (Beijing, China) for cDNA library construction, sequencing, and analysis.

**(ii) Differential expression analysis.** Differential expression analysis (for DESeq with biological replicates) was performed using the DESeq R package (version 1.18.0) (90). DESeq provided statistical routines for determining differential expression in digital gene expression data using a model based on the negative binomial distribution. The resulting *P* values were adjusted using the Benjamini-Hochberg approach for controlling the false discovery rate. Genes with an adjusted *P* value of <0.05 found by DESeq were assigned as differentially expressed.

**(iii) R methods.** Transcriptional groups of interest were manually selected from GO biological process and cellular function groups. Genes possessing GO annotations referring to multiple transcriptional groups were assigned with priority as follows: Iron, Sulfur, Nitrogen, Oxidative Stress, Cellular Damage, and Regulators. Visualization of differential expression using reads per million per kilobase (RPMK) and log2-fold change was done in R (version 4.1.0) using the base and graphics packages.

### qRT-PCR gene expression.

*Rs* cells were grown in 15-mL conical tubes in BD Gaspak anaerobic jars (BD, Franklin Lakes, NJ) for 15 h, then 1 mM SNP or water control was added along with a fresh anaerobic sachet and cultures were grown for a further 3 h under hypoxic denitrifying conditions, as described above. Total RNA was extracted using a hot phenol chloroform method, as described (20). DNA was removed with DNAfree DNase (Invitrogen, Life Technologies, Calrsbad, CA), and cDNA and no-RT controls were synthesized from 200 ng to 1 μg RNA using the SuperScript VILO cDNA synthesis kit (Life Technologies, Carlsbad, CA). The qRT-PCRs were run in triplicate with 5 ng cDNA and Power Up SYBR Green Master Mix (Applied Biosystems, Foster City, CA) in a 10-μL volume using an ABI 7300 Real-time PCR System (Applied Biosystems). Relative gene expression was calculated using the 2^-ΔΔ^*^CT^* method, normalizing to the consistently expressed *rplM* gene (62).

All primer sets amplified fragments between 100 and 200 bp, had 90 to 110% efficiency, and are listed in the supplementary information in Table S5.

### Nitrosative stress assay.

Denitrifying R. solanacearum cells were grown in VDM + 30mM NO_3_^-^ in 96-well microtiter plates in anaerobic pouches (BD, Franklin Lakes, NJ) in a 28°C shaking incubator at 225 rpm. After 16 h, cells were treated with water, 100 μM Spermine-NONOate and water, or 500 μM H_2_O_2_, and returned to the pouches with fresh anaerobic sachets for a further 3 h. After this second incubation, bacterial survival was measured as cell density in a microplate reader (BioTek, Winooski, VT, USA) using Abs_600_. We measured cell density as an indicator of bacterial CFU/mL, as previously validated by dilution plating (7).

### Quantification of intracellular aconitase activity.

*Rs* strains were grown overnight in 5 mL VDM at 28°C, 0% O_2_, and cultures were standardized turbidometrically; the relationship between culture optical density at 600 nm and CFU/mL was previously determined by serial dilution plating (7). About 10^10^ CFU were pelleted and resuspended in water with 20 mg/mL lysozyme (Sigma-Aldrich) to a 5-mL volume, then incubated on ice for 45 min. Cell suspensions on ice were then sonicated with a needle sonicator at 40% amplification for ten 30-s pulse cycles, with 10 s between cycles. The resulting lysates were then used in the aconitase assay (Sigma-Aldrich) in a 96-well plate format, according to the kit instructions. Samples were measured at 450 nm in a microplate reader (BioTek, Winooski, VT, USA) and analyzed to determine units of activity per cell according assay protocol.

### Data availability.

The gene expression data supporting this research are openly available in the Gene Expression Omnibus database at https://www.ncbi.nlm.nih.gov/geo, under the following accession numbers: GSE160024 (R. solanacearum wild-type strain GMI1000, *ΔnorB*, and other mutants growing in culture and in tomato stems) and GSE194210 (whole-transcriptome data for R. solanacearum wild-type strain GMI1000, *ΔnorA*, and *ΔhmpX* growing in culture.
