# Supplementary material for: NorA, HmpX, and NorB Cooperate to Reduce NO Toxicity during Denitrification and Plant Pathogenesis in Ralstonia solanacearum
Source: Microbiol Spectr. 2022 Apr 4;10(2):e00264-22. doi: 10.1128/spectrum.00264-22 (PMC9045102; doi:10.1128/spectrum.00264-22)
Supplement: SUPPLEMENTAL FILE 1 — Supplemental material. Download SPECTRUM00264-22_Supp_1_seq11.pdf, PDF file, 1.3 MB [file spectrum00264-22_supp_1_seq11.pdf]

Supplemental Materials

Truchon AN, CG Hendrich, AF Bigott, BL Dalsing, C Allen*. NorA, HmpX, and NorB cooperate to reduce NO toxicity during denitrification and plant pathogenesis in *Ralstonia solanacearum*. *Corresponding author, callen@wisc.edu

Supplemental Figures

Figure S1. NorA, NorB, and HmpX homologues are conserved across plant and human pathogens, and across Gram positive and Gram negative bacteria. Comparison of *R. solanacearum* NorA, NorB, and HmpX to homologs in *Cupriavidus necator, Nisseria gonorrhoeae, Staphlococcus aureus, Escherchia coli, Salmonella enterica,* and *Xylella fastidiosa.* A. MUSCLE alignment of NorA homologs. **B.** MUSCLE alignment of HmpX homologs. **C.** MUSCLE alignment of NorB homologs. Residues highlighted in pink are involved in iron (Fe^{2+}) binding (Strube et al 2007). Asterisks (*) denote fully conserved residues, a colon (:) indicates conservation between groups of strongly similar properties, and a period (.) denotes conservation between groups of weakly similar properties.

A. NorA

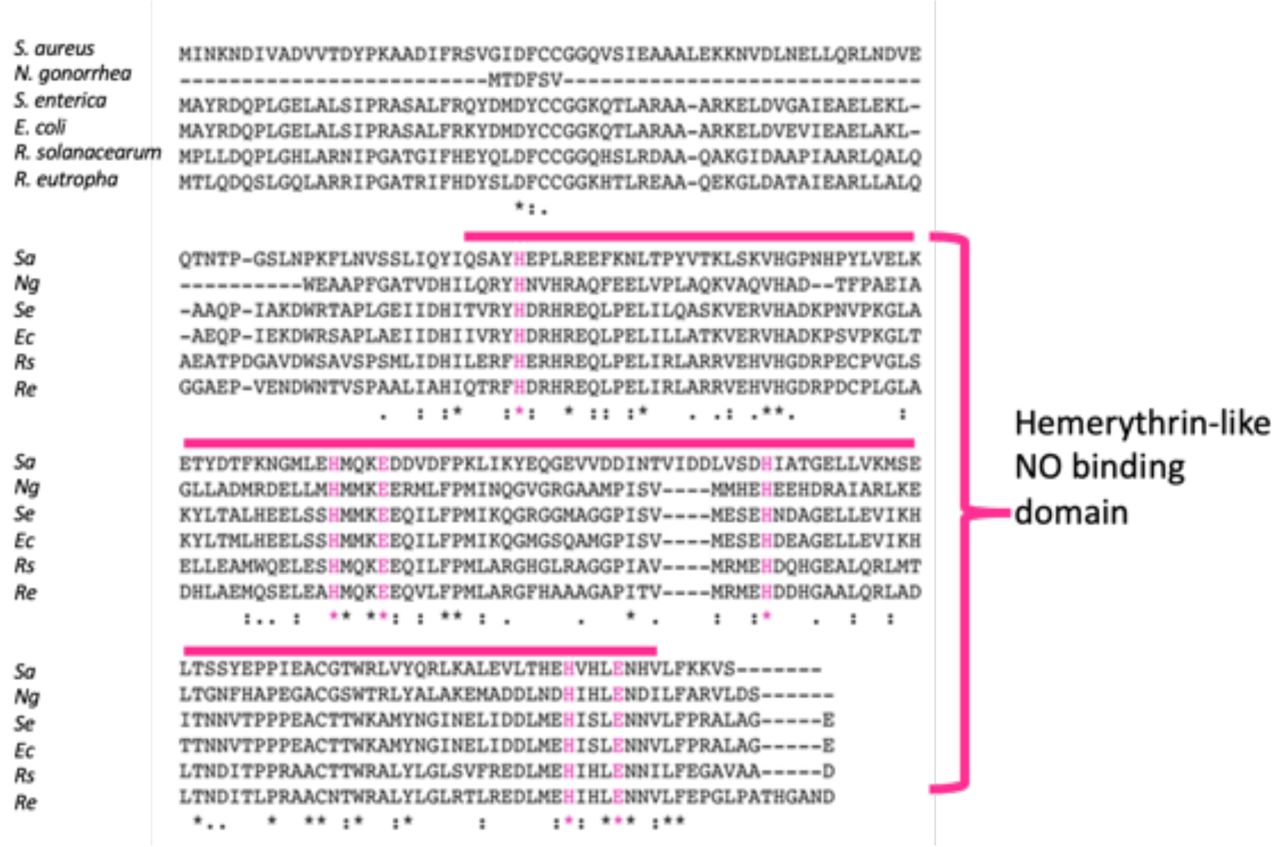

B. HmpX

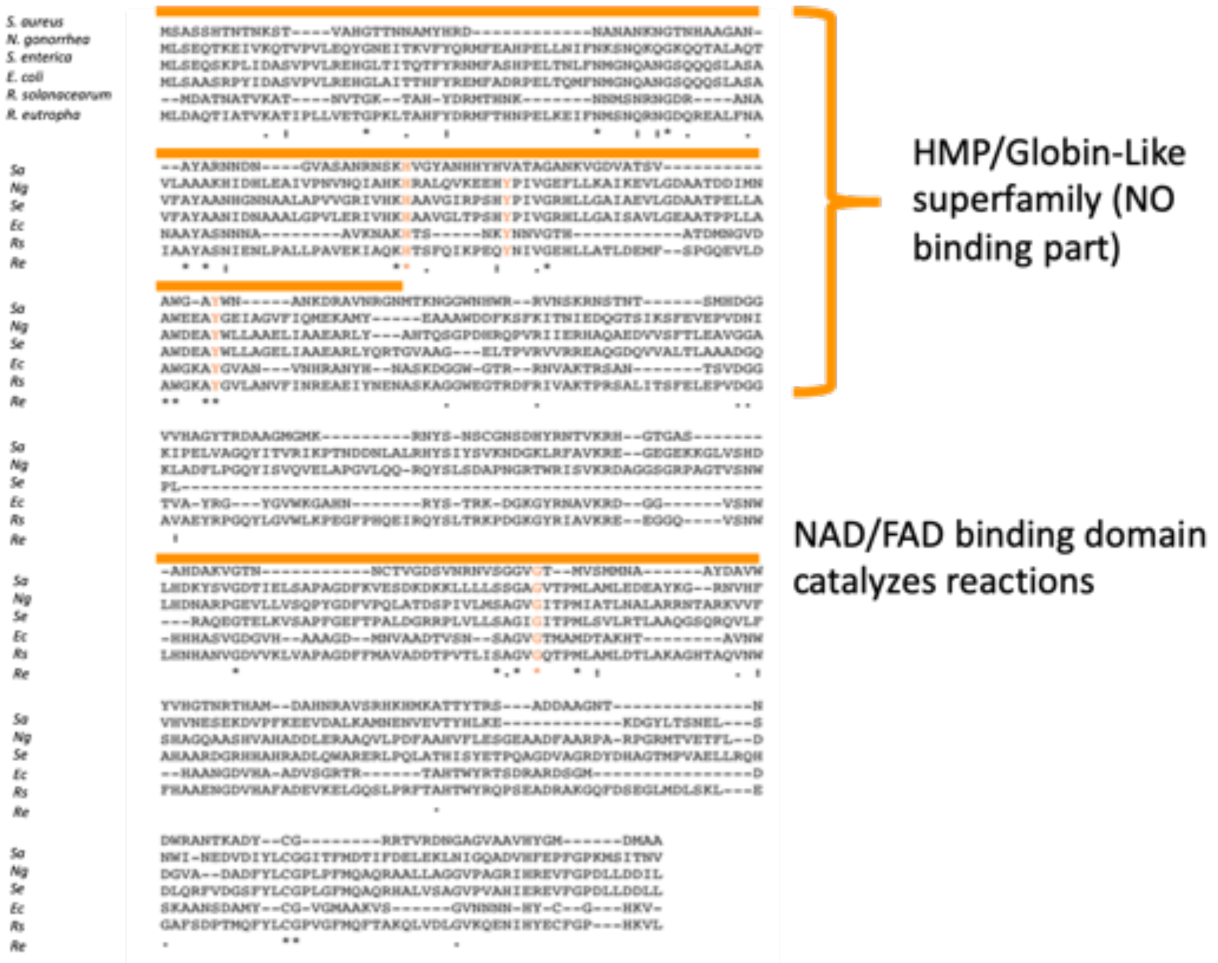

Figure S1. NorA, NorB, and HmpX homologues are conserved across plant and human pathogens, and across Gram positive and Gram negative bacteria. Comparison of *R. solanacearum* NorA, NorB, and HmpX to homologs in *Cupriavidus necator, Nisseria gonorrhoeae, Staphlococcus aureus, Escherchia coli, Salmonella enterica,* and *Xylella fastidiosa.* A. MUSCLE alignment of NorA homologs. **B.** MUSCLE alignment of HmpX homologs. **C.** MUSCLE alignment of NorB homologs. Residues highlighted in pink are involved in iron (Fe^{2+}) binding (Strube et al 2007). Asterisks (*) denote fully conserved residues, a colon (:) indicates conservation between groups of strongly similar properties, and a period (.) denotes conservation between groups of weakly similar properties.

C. NorB

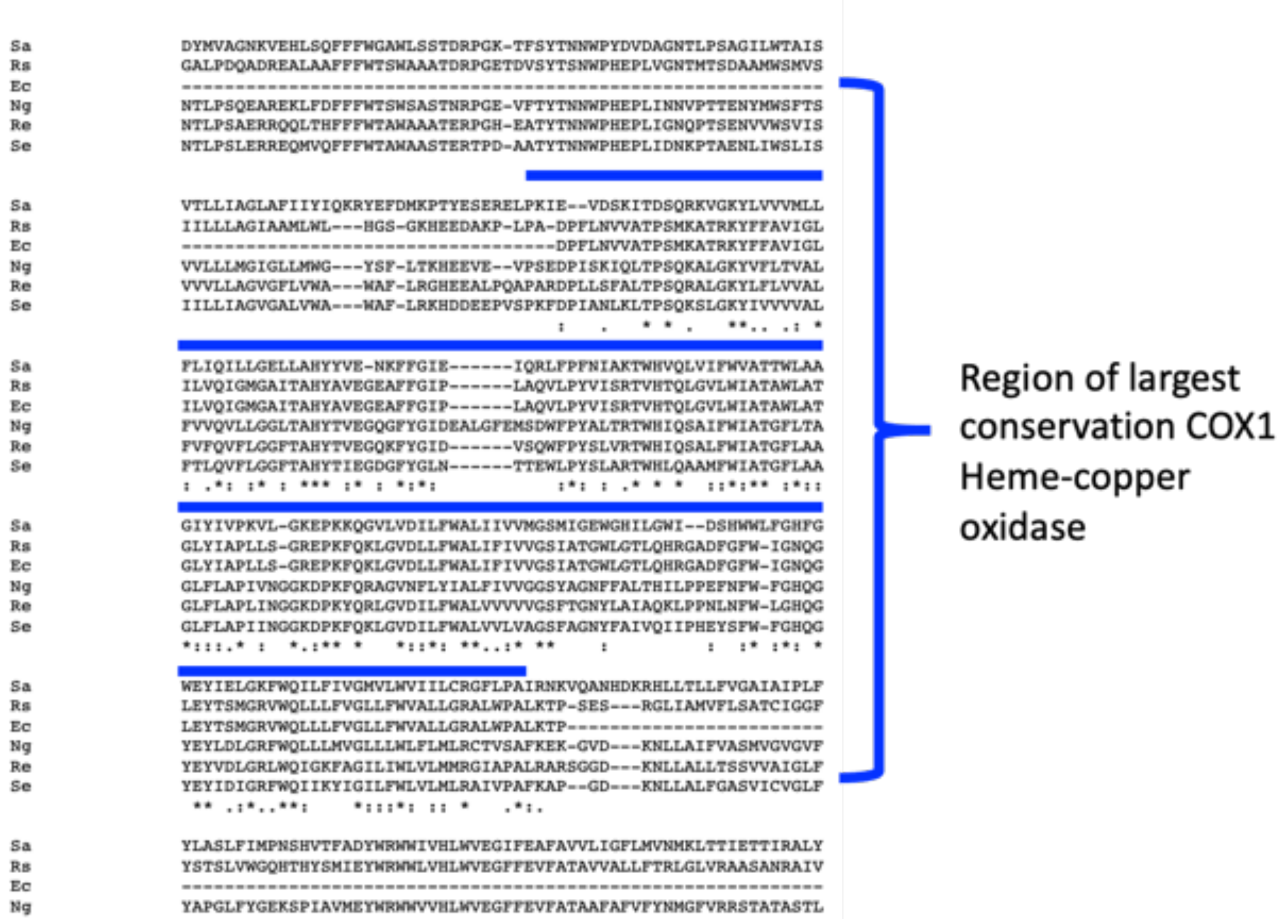

Figure S1. NorA, NorB, and HmpX homologues are conserved across plant and human pathogens, and across Gram positive and Gram negative bacteria. Comparison of *R. solanacearum* NorA, NorB, and HmpX to homologs in *Cupriavidus necator, Nisseria gonorrhoeae, Staphlococcus aureus, Escherchia coli, Salmonella enterica,* and *Xylella fastidiosa.* A. MUSCLE alignment of NorA homologs. **B.** MUSCLE alignment of HmpX homologs. **C.** MUSCLE alignment of NorB homologs. Residues highlighted in pink are involved in iron (Fe^{2+}) binding (Strube et al 2007). Asterisks (*) denote fully conserved residues, a colon (:) indicates conservation between groups of strongly similar properties, and a period (.) denotes conservation between groups of weakly similar properties.

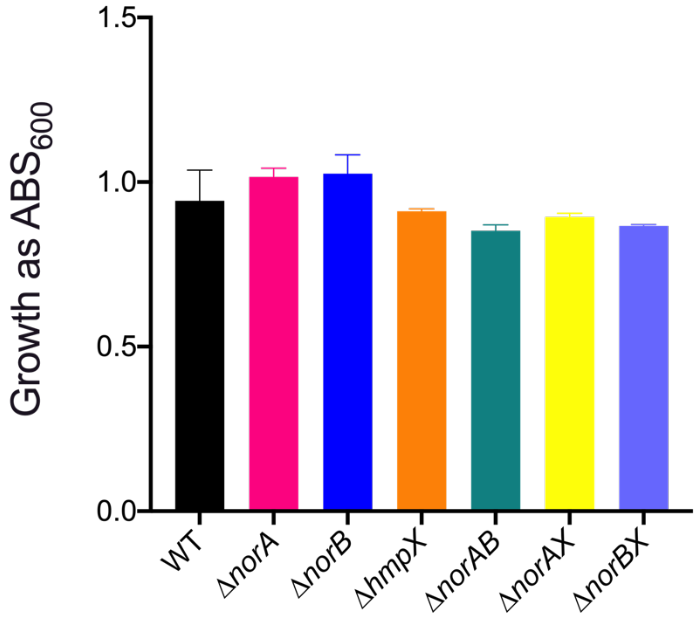

Figure S2. NorR regulates *norA* in *Ralstonia solanacearum*, but not *norB*. A. Relative gene expression of Δ*norR* cells growing in denitrifying VDM + 30mM NO_3^-, 0% O_2) Δ*norR* cells after 16 h growth HPI normalized to *rplM,* relative to WT GMI1000. These data suggest *norA* is the sole gene NorR regulon. Asterisks indicate: * $P < 0.0288$, **** $P = 0.0001$ (one sample t-test) relative to wild-type gene expression.

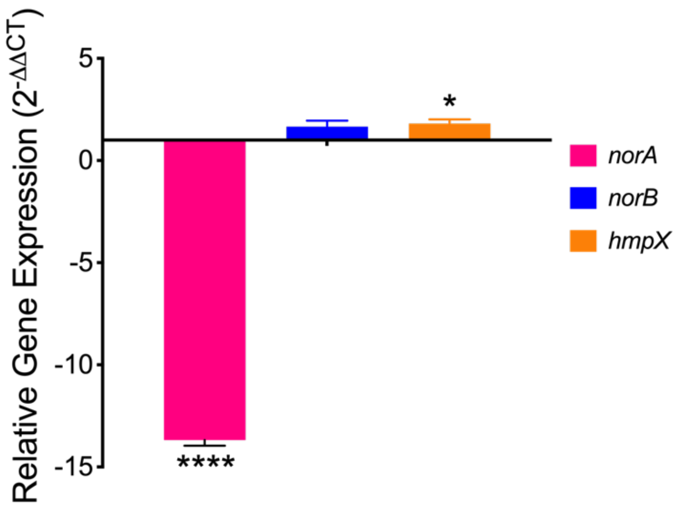

Figure S3. *R. solanacearum* Δ*norA*, Δ*norB*, and Δ*hmpX* mutants grow as well as wild type in aerobic (non-denitrifying) conditions. Endpoint growth of wild type and mutant *Rs* cells growing aerobically in rich media (CPG) in shaking 96 well plates for 36 h, shown as Abs_{600}. Data are mean +/- SEM of 4 biological experiments, each with 3 technical replicates. All strains grew in a manner indistinguishable from that of the wild-type strain GMI1000 (P>0.05, T-test)

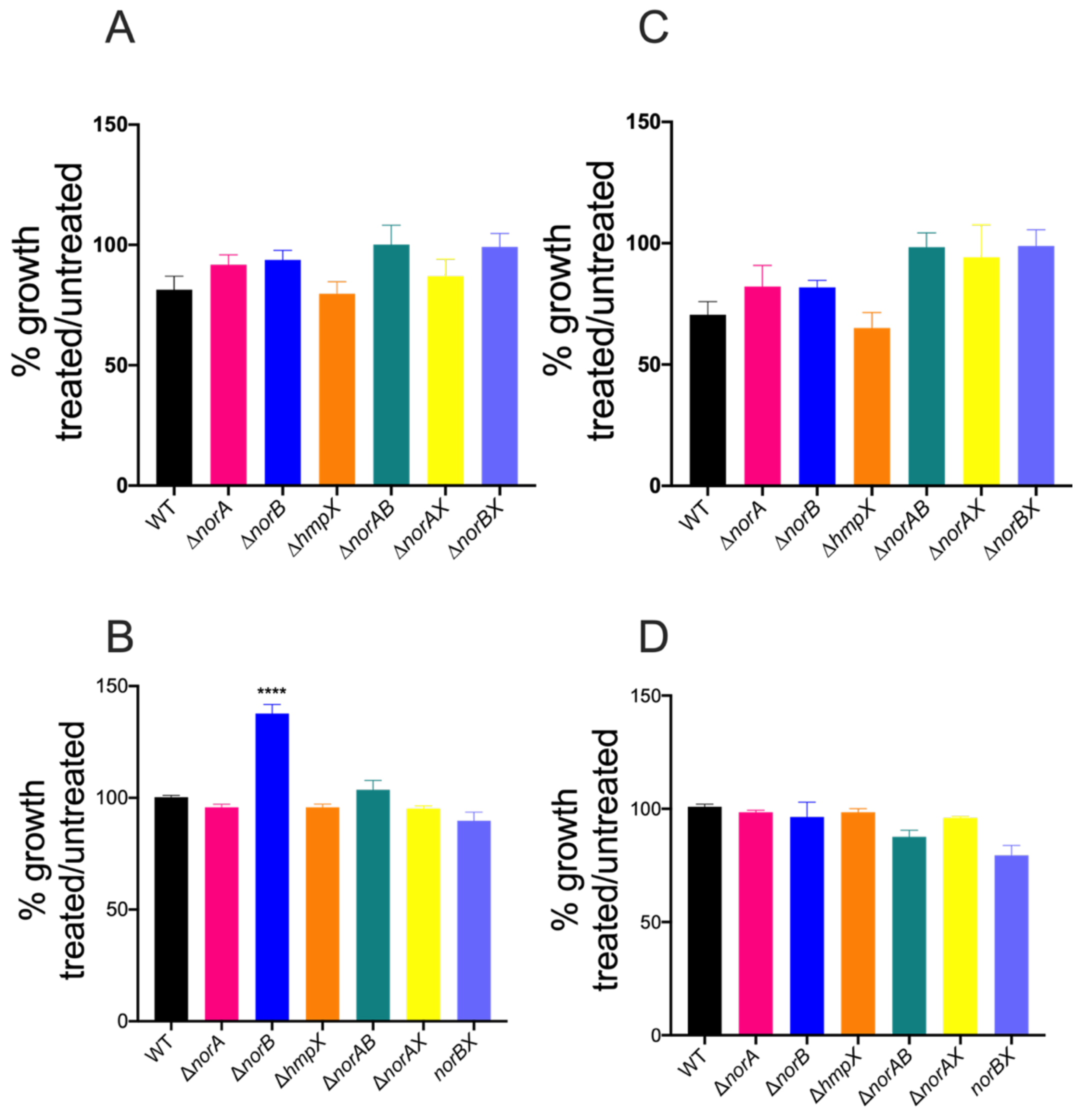

Figure S4. Toxicity of exogenous NO and H_2O_2 treatment on *R. solanacearum* growing in denitrifying conditions. *R. solanacearum* cells were cultured in VDM + 30 mM NO_3^- 0.1% O_2 for 16 h (A and B) or 36 h (C and D) before treatment. **A. and C.** Growth of *Rs* cells treated with 100 µM H_2O_2 measured 4 hours post-treatment and shown as % O.D.$_{600}$ of untreated cells. **B. and D.** Growth of *Rs* cells treated with 100 µM Spermine-NONOate measured 4 hours post-treatment and shown as % O.D.$_{600}$ of untreated cells. Data shown are mean+/- SEM of 3 biological replicates each containing 4 technical replicates. Asterisks represent P < 0.001 (ANOVA).

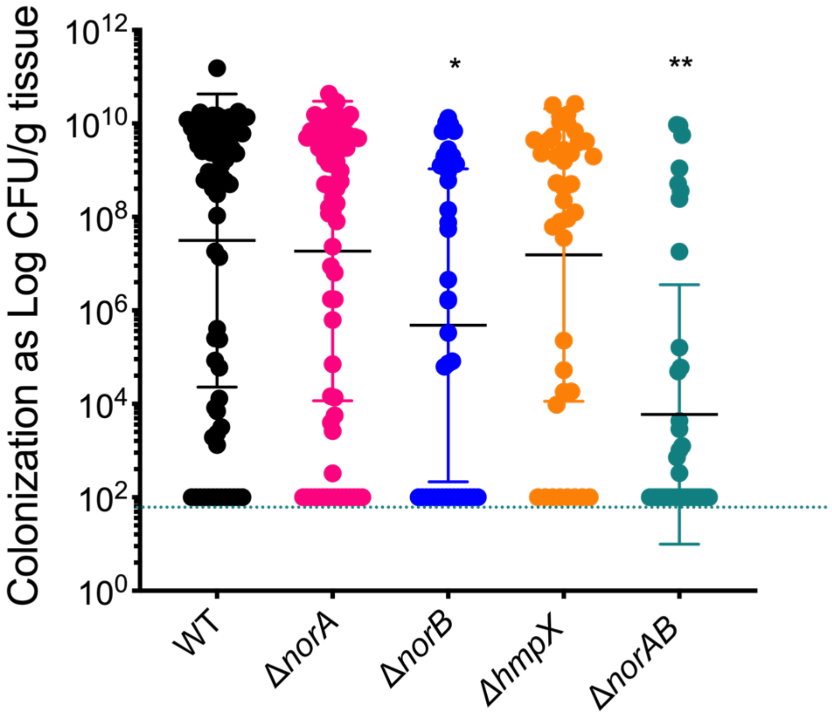

Figure S5. *R. solanacearum* colonization of Bonny Best tomato plants. *R. solanacearum* population sizes in tomato mid-stems 4 days after $2x10^6$ CFU of *Rs* were applied to the cut petiole of the first true leaf. Populations were quantified by grinding and serially dilution plating stem cross sections. Each dot shows the *Rs* population size from one plant, with 40-80 plants per treatment in 2-4 biological replicates. Horizontal bars indicate the geometric mean of the population size; values for stem samples below limit of detection set at limit of detection (100 CFU/mL), asterisks indicate mean population size is different from wild type (*P < 0.0049,**P < 0.0001, Mann-Whitney test).

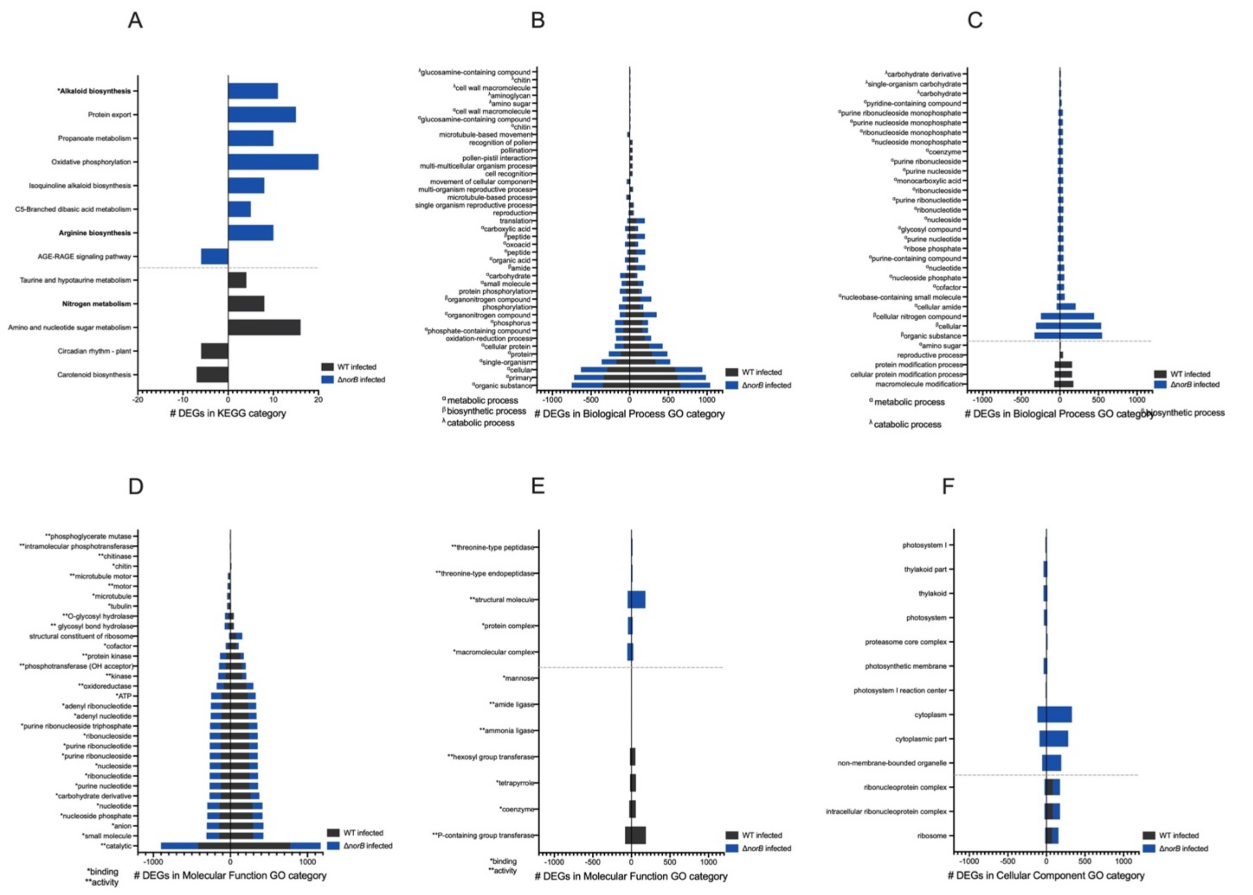

Figure S6. Transcriptomic response of tomato plants infected with either *R. solanacearum* wild-type strain GMI1000 or the Δ*norB* mutant. RNA was harvested and sequenced from stems of 'Bonny Best' tomato plants 72 h after they were petiole-inoculated with $2x10^6$ CFU of either wild-type, Δ*norB* cells or water (healthy control). Wild-type or Δ*norB* infected plants were compared to water-inoculated plants to identify differentially expressed genes (DEGs). The X axis shows the number of differentially expressed genes (DEGs) assorted into the indicated KEGG (A) or GO categories (B-F), as determined with KOBAS (KEGG) or GO database, http://www.geneontology.org/ by NovoGene. For details on identification of DEGs, see methods. Number of DEGs in each KEGG or GO term categories from wild-type or Δ*norB* infected plants graphed in stacked columns for comparison. **A.** 5 KEGG categories for DEGs unique to wild-type and 8 KEGG categories for DEGs unique to Δ*norB* infected plants. * indicates Tropane, piperidine, and pyridine alkaloid biosynthesis. **B**. DEGs were sorted into 41 Biological Process GO terms shared between wild-type or Δ*norB* infected plants. **C.** 5 Biological Process GO terms were unique to wild-type infected plants and 28 were unique to Δ*norB* infected plants. **D.** 32 Molecular Function GO terms are shared between wild-type or Δ*norB* infected plants. **E.** 7 Molecular Function GO terms were unique to wild-type infected plants and 5 were unique to Δ*norB* infected plants. **F.** 3 Cellular Component go terms are shared between wild-type and Δ*norB* infected plants. Ten Molecular Function GO terms were unique to Δ*norB* infected plants but there were no terms unique to wild-type infected plants. Biological Process and Molecular Function GO terms, terms simplified by abbreviating metabolic process (α), biosynthetic process (β) catabolic process (γ), binding (*), and activity (**). Broad GO term categories biological process, and metabolic process, and catalytic activity were removed for graph clarity.

Supplemental Tables

Supplemental Table 1A: Traits of NorA, NorB, and HmpX homologs from other bacteria

	NorA homologs				NorB homologs				HmpX homologs			
**Species**	**Protein**	**Length (AA)**	**% AA ID**	**% QC**	**Protein**	**Length (AA)**	**% AA ID**	**% QC**	**Protein**	**Length (AA)**	**% AA ID**	**% QC**
C. necator	NorA	227	72	99	NorB	752	48	98	flavohemeprotein	346	50	100
N. gonorrhoeae	DnrN	157	45	65	NorB	746	44	97	-	-	-	-
S. aureus	ScdA	224	32	94	COX1	752	35	97	flavohemeprotein	385	35	100
S. enterica	YtfE	220	51	100	NorB	726	48	95	hmpA	273	20	31
X. fastidiosa	-	-	-	-	-	-	-	-	hmpA	289	25	66

Amino acid (AA) sequence of *Ralstonia solanacearum* NorA, NorB, and HmpX was blasted in NCBI for homology to other known pathogens to find %AA identity (%AAID) and % query cover (QC).

Supplemental Table 1B: Gene expression of *norA*, *norB*, and *hmpX* in culture (CPG= rich medium, aerobic or VDM = denitrifying conditions, 0.01% O_2) and *in planta (ip* = in tomato xylem)

	WT *in vitro* vs *in planta*		Mutants vs WT VDM		
gene	CPG vs *ip*[JJ]	VDM vs *ip**	Δ*norA* vs WT*	Δ*norB* vs WT*	Δ*hmpX* vs WT*
norB	**51**	**20.7551866**	1.19520658	***-1.3457776***	**-1.6128363**
hmpX	**43**	**5.71875064**	**-1.9637347**	**11.6673094**	***-5.7139959***
norA	**75**	**44.461971**	***-126.42167***	**2.43749625**	-1.2784095

Fold change gene expression data, negative values (less than 1) shown as negative reciprocal (value/-1), Bolded numbers are significant DEGs
ip = *in planta,* jj = Jacobs 2012*,* * = this study

Supplemental Table 2: Differential Bacterial Gene Expression Table (separate Excel Sheet)
Raw bacterial gene expression data for *R. solanacearum* wild type, Δ*norA*, Δ*norB*, Δ*hmpX* cells growing in denitrifying media and growing in infected Bonny Best tomato plants (for wild type and Δ*norB*).

Supplemental Table 3: Differential Plant Gene Expression Table (separate Excel Sheet)
Raw plant gene expression data for wilt-susceptible tomato *cv.* Bonny Best infected with water (control), *R. solanacearum* wild-type strain GMI1000 or the Δ*norB* mutant.

Supplemental Table 4: Strains, plasmids, and primers used in this study

Strain, Plasmid, Primer	Locus Relevant characteristics /Sequence 5' to 3'	Function	Ref
***Rs* strains**			
GMI1000	Wild-type; phylotype I strain		(6)
Δ*norA*	GMI1000 Δ*norA*	putative di-iron binding protein	This study
Δ*norR*	GMI1000 Δ*norR*	NO responsive regulator	This study
Δ*norB*	GMI1000 Δ*norB*::Gm; Gm^r	NO reductase	(7)
Δ*hmpX*	GMI1000 Δ*hmpX*::Gm; Gm^r	NO oxidoreductase	(7)
Δ*norAB*	GMI1000 Δ*norAnorB*::Gm; Gm^r		This study
Δ*norAhmpX*	GMI1000 Δ*norAhmpX*::Gm; Gm^r		This study
Δ*norABhmpX*	GMI1000 Δ*norAnorBhmpX*::Gm; Gm^r		This study
**Plasmids**			
pUFR80	pUFR80 sacB – kanR - Cloning plasmid		(48)
**Primers**			
norA	F - CCAGCATCGGAAACAGGATCT	putative NO-binding protein	This study
	R - CGGTTGGCTTGAGCGAGTT		
norB	F -CACACCCACTACTCGATGAT	nitric oxide reductase	This study
	R -AACAGGAACACGATGGTCTC		
hmpX	F - CATCACCCAGACCTTCTACC	nitric oxide oxidoreductase	This study
	R - CATGCTTGTGGACGATGC		
rplM	F - CCGCAAAGCCCCATGA	50s ribosomal subunit	(53)
	R - TGTCCGTCGCGTCAATCA		
Gib *norA* down	F-CAGGTCGACTCTAGAGCTTCCTGATCTCCGATGAACGGGGAGGACGC		This study
	R - TGGACCAGGTGCGTGGCGGGCGGGCG		
Gib *norA* up	F - ACCACGCACCTGGTCCAGTAACGGCATTGCATGCTCCTTTGTG		This study
	R - TAGCTCGGTACCCGGGGTGCCGCCGTCGGCGGCC		
Gib *norR* up	F - TCGTGAAAGCGCTCGAGAAT		This study
	R - CAGGAGCAGACGGTCAATCA		
Gib *norR* down	F - GCAGGCATGATGGACCGTG		This study
	R - GCCCTATACCTGCTTCCTCG		
